# Distribution-based measures of tumor heterogeneity are sensitive to mutation calling and lack strong clinical predictive power

**DOI:** 10.1038/s41598-018-29154-7

**Published:** 2018-07-30

**Authors:** Javad Noorbakhsh, Hyunsoo Kim, Sandeep Namburi, Jeffrey H. Chuang

**Affiliations:** 1 The Jackson Laboratory for Genomic Medicine, Farmington, CT USA; 2 University of Connecticut Health Center, Department of Genetics and Genome Sciences, Farmington, CT USA

## Abstract

Mutant allele frequency distributions in cancer samples have been used to estimate intratumoral heterogeneity and its implications for patient survival. However, mutation calls are sensitive to the calling algorithm. It remains unknown whether the relationship of heterogeneity and clinical outcome is robust to these variations. To resolve this question, we studied the robustness of allele frequency distributions to the mutation callers MuTect, SomaticSniper, and VarScan in 4722 cancer samples from The Cancer Genome Atlas. We observed discrepancies among the results, particularly a pronounced difference between allele frequency distributions called by VarScan and SomaticSniper. Survival analysis showed little robust predictive power for heterogeneity as measured by Mutant-Allele Tumor Heterogeneity (MATH) score, with the exception of uterine corpus endometrial carcinoma. However, we found that variations in mutant allele frequencies were mediated by variations in copy number. Our results indicate that the clinical predictions associated with MATH score are primarily caused by copy number aberrations that alter mutant allele frequencies. Finally, we present a mathematical model of linear tumor evolution demonstrating why MATH score is insufficient for distinguishing different scenarios of tumor growth. Our findings elucidate the importance of allele frequency distributions as a measure for tumor heterogeneity and their prognostic role.

## Introduction

A major challenge for predicting clinical outcome to cancer treatment is the heterogeneity of cell populations within each tumor, as intratumoral heterogeneity is increasingly being associated with metastasis and resistance to therapies^1–4^. Intratumoral heterogeneity develops from mutations in cells and the relative growth advantages they confer to their descendant populations^5,6^. To better understand this phenomenon, multiple research groups have developed methods for the challenging problem of estimating tumor cellular composition from bulk tumor sequencing data^7–13^. These methods rely on the allele frequencies of mutations in each cancer, with low allele frequency mutations providing information on small subclones and high allele frequency mutations providing information on large subclones. Factors such as ploidy, copy number, and tumor purity can also affect this inference. In particular, aneuploidy in DNA mixtures has been associated with poor subclonal inference^14^. Still, accurate measures of allele frequencies are central for evaluating subclonal heterogeneity and its clinical implications.

Increased heterogeneity has been theorized to lead to worse patient survival due to the increased potential for resistant populations^15–17^, but it remains unclear if current measures of tumor heterogeneity are sufficient to resolve such an effect. Prior studies have attempted to determine this relationship. For example, Rocco *et al*.^18^ used MATH score, a measure of the width of the allele frequency distribution, as a proxy for tumor heterogeneity and observed poorer survival for head and neck squamous cell carcinoma (HNSC) in patients with higher MATH score. More recently, Morris *et al*.^19^ found associations between heterogeneity and survival in several cancers using a multivariate regression of MATH and other variables versus survival. However, a key caveat is that all heterogeneity measures, including MATH, are affected by the accuracy of mutation calls. Many studies have shown that cancer mutation calls can differ substantially depending on the algorithm used for their determination^20–23^. In order to verify if heterogeneity impacts survival, it is necessary to quantify the robustness of allele frequency distributions to mutation callers as well as the robustness of their relationship to survival.

Allele frequency distributions also provide information on the evolutionary processes in tumors, which remain poorly understood. While a variety of intratumoral evolutionary models have been proposed^24–27^, the impact of allele frequency accuracy on evolutionary inference has not been substantially explored. Determining the robustness of allele frequency distributions will elucidate this problem.

In this paper we study the robustness of allele frequency distributions and their relevance to patient survival. To the best of our knowledge this is the first study which explores sensitivity of these distributions and their clinical prognostic power to different mutation calling methods. We call single nucleotide variations (SNVs) from 11 cancer types in The Cancer Genome Atlas (TCGA) using three common mutation callers: MuTect^28^, SomaticSniper^29^, and VarScan^30,31^. To determine if the resulting allele frequency distributions are clinically useful, we analyze the correlation between these distributions and patient survival. Our study demonstrates whether allele frequency variability is clinically predictive and what other genomic features mediate these results. Finally, we discuss implications of a simple evolutionary mechanism on allele frequency distributions using a mathematical modeling approach.

## Results

### Different mutation callers lead to distinct allele frequency distributions

**Figure 1:**
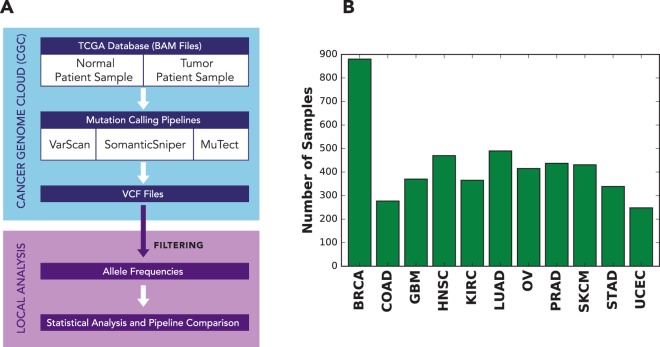
Analysis design. (**A**) Schematic of the analysis process for 4722 TCGA samples. Somatic mutation calling was carried out on the NCI Cancer Genomics Cloud^33^, followed by additional local analysis. (**B**) Number of samples studied from each cancer.

We analyzed a total of 4722 samples from the TCGA database on a cloud computing platform to explore the effects of mutation calling by MuTect, SomaticSniper, and VarScan on allele frequency distributions and patient survival. Tumor/normal matched BAM files that had been aligned to the hg19 reference were used to call the SNVs (Fig. 1A). The mutation calling pipelines (Fig. S1) were implemented by dockerizing^32^ each element of the pipeline and linking them through an interface provided by the Cancer Genome Cloud (CGC)^33^. Three different mutation calling pipelines were run for each tumor/normal pair on Amazon Web Services (AWS)^34^ through the CGC interface, and the resulting allele frequencies were calculated for each sample.

To analyze the range of possible allele frequency behaviors, we chose 11 cancer types from the TCGA database (Fig. 1B and Fig. S2), namely breast invasive carcinoma (BRCA), colon adenocarcinoma (COAD), glioblastoma multiforme (GBM), head and neck squamous cell carcinoma (HNSC), kidney renal clear cell carcinoma (KIRC), lung adenocarcinoma (LUAD), ovarian serous cystadenocarcinoma (OV), prostate adenocarcinoma (PRAD), skin cutaneous melanoma (SKCM), stomach adenocarcinoma (STAD), and uterine corpus endometrial carcinoma (UCEC).

We found that overall MuTect called the largest number of SNVs and VarScan the least. On average, each sample called by MuTect had 1.4 times more SNVs compared to VarScan and 1.3 times compared to SomaticSniper (Fig. S3). On average, VarScan, SomaticSniper, and MuTect respectively called 1.5, 1.8, and 2.3 times more SNVs compared to the consensus SNVs (i.e. those called by all three mutation callers).

**Figure 2:**
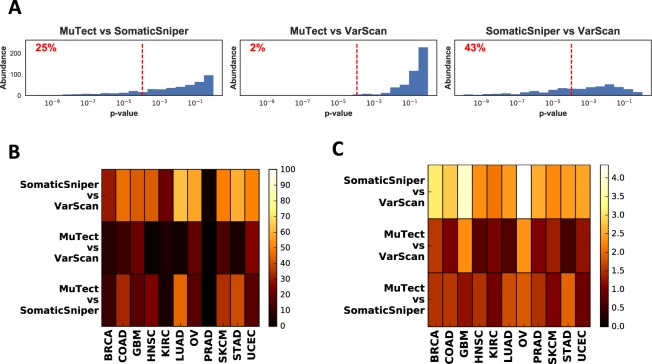
Comparisons of allele frequency distributions for different mutation callers. (**A**) Distribution of Kolmogorov-Smirnov (KS) test p-values corresponding to the null hypothesis that mutation callers will produce similar allele frequency distributions, plotted pairwise for HNSC. The red dashed line indicates the Bonferroni corrected significance threshold $\alpha =0.05$. The percentage of samples that fall below this threshold are shown on each graph. (**B**) Percentage of significantly different samples for mutation caller pairs, grouped by cancer type. (**C**) Comparison of allele frequency distributions using earth mover’s distance.

We then compared allele frequency distributions assessed by pairs of mutation callers using the Kolmogorov-Smirnov (KS) test (Fig. 2A,B). Distributions were not very robust across different cancer types (Fig. 2B). SomaticSniper and VarScan produced the most dissimilar allele frequency distributions with 42 ± 14% (mean ± std) of samples being significantly different across cancers. MuTect and VarScan produced the least dissimilar results with 11 ± 8% of samples significantly different. This percentage was 22 ± 14% when MuTect was compared against SomaticSniper. Prostate adenocarcinoma (PRAD), showed unusually robust results, with only 1%, 1%, and 3% of samples being significantly dissimilar when MuTect-SomaticSniper, MuTect-VarScan, and SomaticSniper-VarScan were compared pairwise, respectively.

Due to sensitivity of the Kolmogorov-Smirnov test to the number of SNVs in the tumor (Fig. S4), we also used the earth mover’s distance (EMD) to assess differences between allele frequency distributions. EMD produced qualitatively similar results (Fig. 2C) to the Kolmogorov-Smirnov test, with SomaticSniper-VarScan showing the most difference among all pairwise comparisons and MuTect-VarScan showing the least difference. Another method based on cumulative absolute differences between pairs of distributions as well as a non-parametric test based on quadratic differences of distributions^35^ also yielded similar results (Fig. S5, and *Methods*). Across all cancers, per-sample correlation coefficient between allele frequencies of shared mutations was 0.97 ± 0.1, regardless of which pair of mutation callers were compared. Hence, differences between mutation caller results tend to be caused by disagreement about mutation calls rather than allele frequencies.

Copy number variations (CNV) can influence allele frequencies and may indirectly shape their distribution. To assess this effect, we repeated the analysis after removing somatic SNVs with copy number aberrations (|*CNV*| > 0.2). Here and throughout the text we use the *CNV* quantity to denote ${\mathrm{log}}_{2}(\frac{{\rm{copy}}\,{\rm{number}}}{2})$, as provided by TCGA (see *Methods*). Comparison of allele frequency distributions using Kolmogorov-Smirnov statistics (Fig. S6) did not produce qualitatively different results from those shown in Fig. 2B, and again showed the most pan-cancer differences between SomaticSniper and VarScan. These results suggest that copy number impacts all mutation callers similarly.

### MATH score is a poor predictor of patient survival across cancer types

To investigate the relationship of tumor heterogeneity and survival, Mroz *et al*.^18^ introduced a measure of heterogeneity they term MATH. It is the ratio of scaled median absolute deviation (MAD) to median stated in percentage:

1 $${\rm{MATH}}=100\times \frac{{\rm{MAD}}}{{\rm{Median}}}$$

We calculated this measure for all samples and mutation callers. Overall VarScan yielded higher median MATH score (35.4 ± 5.7) compared to SomaticSniper (24.4 ± 4.8) and MuTect (30.3 ± 5.9) (Fig. S7). Similar to Fig. 2, the MATH scores were also more similar between MuTect and SomaticSniper calls (pan-cancer Spearman correlation coefficient *r* = 0.7 ± 0.1) and more dissimilar between SomaticSniper and VarScan (*r* = 0.5 ± 0.2) (Fig. S7).

**Figure 3:**
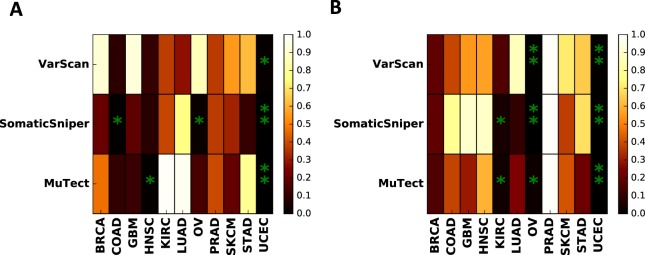
Significance analysis of measures of genomic variations and patient survival. (**A**) Survival analysis log rank test p-values for high and low MATH score groups, and (**B**) Survival analysis log rank test p-values for high and low CNV standard deviation groups. Stars corresponds to values smaller than significance threshold 0.05. Double stars show significant results after Benjamini-Hochberg correction across all cancer types and mutation callers.

We then analyzed the relationship between MATH score and patient survival by grouping samples into high or low MATH groups as compared to the median of the cohort. These two groups were then compared using a log-rank test to determine the significance of MATH values on Kaplan-Meier patient survival curves (Fig. 3 and Fig. S8).

Among the cancers studied, COAD, OV, HNSC, and UCEC showed a significant (*p* < 0.05) relationship between MATH and survival for at least one caller (e.g. COAD p = 0.07, OV p = 0.02 with SomaticSniper calls). However, only UCEC showed a significant relationship for all three mutation callers (p = 0.00083, 0.0023, 0.011 for MuTect, SomaticSniper, and VarScan calls, respectively). Although these results suggest some clinical predictive power of MATH score, a more conservative approach would be to correct for multiple hypothesis testing. Using the Benjamini-Hochberg correction for all cancers and mutation callers led to significant results only for UCEC, and only when the calls were made by MuTect or SomaticSniper. Therefore the predictive power of MATH score is not robust in a pan-cancer analysis except for possibly UCEC.

Previous studies^18,19^ have shown a significant relationship between MATH score and patient survival in HNSC. We obtained similar results when the calls were made using MuTect; however the effect was not significant when other callers were used (Fig. S8). Restricting the analysis to the same samples as in^19^ did not affect the significance of this result (Fig. S9). Thus the clinical predictive power of MATH in HNSC appears to be substantially impacted by the mutation calling method.

To determine the direction of effect of MATH score on survival, we calculated the average survival difference between high and low MATH score groups (*see Methods*). This analysis indicates that for UCEC higher MATH score is associated with poorer patient survival (Fig. S10). The same trend is observed for COAD and HNSC. For OV, the direction of the trend is caller-dependent.

### Copy number mediates prognostic power of allele frequency distributions

Allele frequency variation in a tumor is impacted by locus-to-locus copy number variation in addition to subclonal heterogeneity, and decomposing these two contributions may be important for clinical prognosis. We therefore repeated the above survival analysis but replaced MATH score with the standard deviation of CNV over mutated loci (Fig. 3B and Fig. S11). This CNV analysis yielded more robust results across mutation callers than the MATH score analysis (Fig. S12). Specifically, we found that copy number variation is correlated with survival in KIRC, OV, and UCEC for at least two mutation callers. Multiple hypothesis correction led to a robust effect in UCEC for all mutation callers as well as significance in OV for SomaticSniper and VarScan. This result indicates that copy number variation is a more robust predictor of patient survival than allele frequency distribution.

Despite the lack of statistical significance, for most cancers, higher CNV standard deviation led to poorer survival and this effect was robust across mutation callers (Fig. S10). This was most pronounced for SKCM, STAD, and UCEC. An exception was OV where higher copy number standard deviation was predictive of better survival.

**Figure 4:**
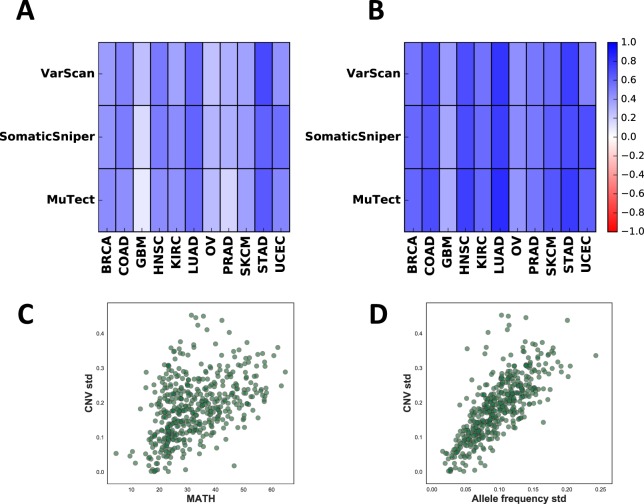
Correlation between allele frequency variations and copy number variations. (**A**) Pearson correlation coefficient of CNV standard deviation and MATH score. (**B**) Pearson correlation coefficient of CNV standard deviation and allele frequency standard deviation. (**C**) CNV standard deviation versus MATH score, plotted for HNSC (SNVs called by MuTect). Each circle is an individual sample. (**D**) CNV standard deviation versus allele frequency standard deviation, plotted for HNSC (SNVs called by MuTect).

To further distinguish the effect of allele frequencies and copy numbers on patient survival, we filtered out mutant allele frequencies at loci with |*CNV*| > 0.2 and calculated the log rank test for MATH using the remaining sites (Fig. S13). Analysis of this set with multiple hypothesis correction did not yield any significant results, indicating that MATH clinical predictions are indeed driven by copy number aberrations. This result can be more clearly seen from the correlation between allele frequency variation and copy number variation. Pearson correlation coefficients between copy number standard deviation and MATH score were positive for all tumor types and all mutation callers (Fig. 4A). This effect was even stronger when we compared copy number standard deviation to allele frequency standard deviation (Fig. 4B). This further supports that allele frequency distributions are highly influenced by copy number aberrations across the genome (see examples in Fig. 4C,D).

To explore how the effect of copy number variation on phenotype is confounded by SNVs, we analyzed gene expression in UCEC (Fig. S14 and *Methods*). The expression levels of most genes (85%, Wilcoxon signed-rank test *p* = 9e−8) containing both SNVs and copy number amplifications was higher than genes lacking both changes, even after discarding tumor-suppressors and oncogenes (84%, *p* = 3e−6). However, amplified genes with SNVs were only slightly over-expressed compared to amplified genes without SNVs and this result was not statistically significant (60%, *p* = 0.38). This suggests that SNVs have a very minor effect on copy number amplification phenotype.

To fully decouple the effects of SNVs and CNVs, we calculated the whole genome CNV standard deviation (WG CNV std) by uniformly sampling the genome for copy number variations and calculating their corresponding standard deviations (see *Methods*). We found that WG CNV std is highly correlated with CNV std (Spearman correlation 0.93 for UCEC; Fig. S14), indicating an almost uniform distribution of SNVs across the genome. Similar to CNV std, increased WG CNV std also led to better patient survival in OV (log-rank *p* = 0.0009) and worse patient survival in UCEC (log-rank *p* = 0.0097), with the former showing significant results after multiple hypothesis correction (see Fig. S14). Overall, these results suggest that SNVs have a minor confounding effect on the clinical prognostic power of global copy number variation.

### Distribution-based measures of intratumoral heterogeneity are consistent with many evolutionary scenarios

**Figure 5:**
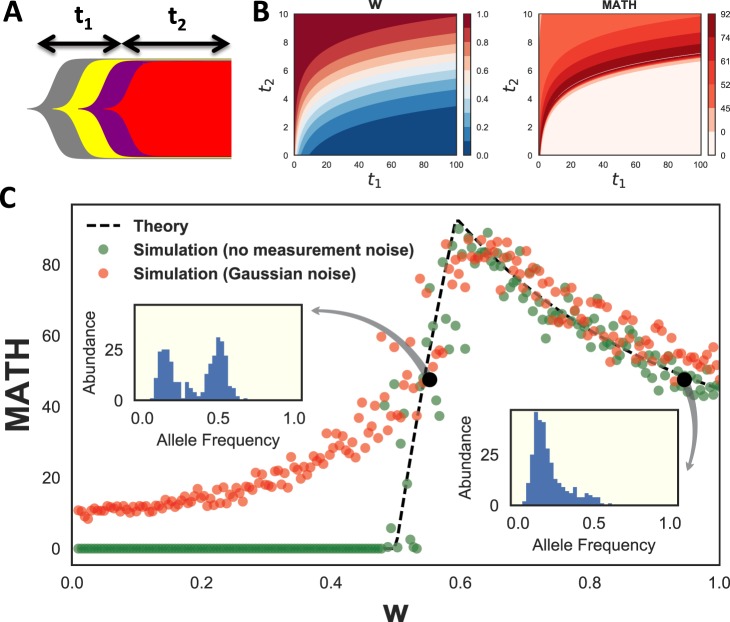
Theoretical analysis of MATH score. (**A**) Schematic diagram of linear tumor evolution model. ${t}_{1}$ is the time from the common ancestor of all tumor cells until the last selective sweep. ${t}_{2}$ is the time from the last selective sweep until biopsy. (**B**) Contour plots of neutral fraction, $w$, and MATH as a function of ${t}_{1}$ and ${t}_{2}$ for a noiseless exponentially growing model of linear evolution. $w$ is the fraction of total SNVs that occurred after the last selective sweep. For this growth model, $w={2}^{{t}_{2}}/({t}_{1}+{2}^{{t}_{2}})$, where time is measured in cell cycles. (**C**) MATH score as a function of neutral fraction, $w$, for a linear tumor evolution model. Dashed lines correspond to analytical calculations. Green dots are samples from the model with 300 SNVs. Red dots are simulated results after incorporation of Gaussian measurement noise. Insets correspond to simulated allele frequency distributions for the two black circles, which have equal MATH scores.

The multiple contributions to MATH suggest a theoretical question: what is the uniqueness of MATH score in distinguishing different underlying evolutionary scenarios? To answer this we considered the allele frequency distributions in the linear evolution model^36^ and computed MATH score as a function of model parameters, in the simplifying case of no CNVs. The linear model is the simplest model of tumor evolution with selection and assumes that occasional driver mutations lead to selective sweeps in a background of neutral mutations (Fig. 5A).

The allele frequency distribution can be specified by a variable *w* that we call the ‘neutral fraction’ (See Methods, eq. (9)), i.e. the fraction of total somatic mutations that occurred after the last selective sweep. We analytically derived a closed form for MATH score as a function of *w* (eq. (10)). Assuming exponential growth for time-dependence, there are a continuous set of choices of *t*_1_ (the time from the common ancestor of all tumor cells until the last selective sweep) and *t*_2_ (the time since the last selective sweep) that lead to identical values of *w* and MATH (Fig. 5B). Thus MATH score does not uniquely specify a tumor’s evolutionary history, even in the absence of copy number effects.

The model presented above is a simplification of one evolutionary scenario and can be influenced by several factors including mutation rate, proliferation rate and cell death. The derivation of eqs 9 and 10 assumes constant mutation rate per cell division after the last selective sweep, but does not put any restrictions on the form of the growth model, as long as the genotype and phenotype remain uncoupled^37^. Any global increase in growth rate or decrease in death rate after the last selective sweep is equivalent to an increase in the neutral fraction, *w*, and does not influence the model otherwise. On the other hand, changes in mutation rates can completely break down these derivations. One exception, however, is if the mutation rate changes during the last selective sweep and remains constant afterwards. An increase of mutation rate in this scenario would not violate model assumptions and is equivalent to an increase in the neutral fraction. Such a scenario may be relevant to some cancer types such as GBM^38^.

To understand the functional dependence of MATH on neutral fraction, we plotted it against *w* and compared it to simulations (Fig. 5C). These results show two regimes of behavior across *w*. MATH score is zero when *w* ≤ 0.5 (the clonal regime – most mutations are clonal due to the sweep). Addition of measurement noise leads to an increase in MATH score in this clonal regime, but does not significantly affect MATH for *w* > 0.5 (the neutral regime – most mutations have evolved neutrally but vary in when they arose). In the neutral regime MATH is not a one-to-one function of *w*, and two very different allele frequency distributions can have the same score. These results show the limitations of single distribution-based scores to uniquely distinguish tumor evolutionary scenarios. Further features of the distribution, such as bimodality, should be used to resolve this degeneracy.

## Discussion

In a pan-cancer analysis of 4722 samples we explored the robustness of allele frequency distributions to mutation calling and their power for clinical prediction. We demonstrated that the mutation calling process can significantly influence the shape of allele frequency distributions. In general, higher variation in allele frequency was correlated with poorer survival, though this result was statistically significant in only a few cases. The only cancer type with statistically significant results robust across mutation callers was UCEC, but this reflected higher levels of copy number variation along the genome rather than increased subclonal heterogeneity. These results suggest that current single-variable measures of subclonal diversity from exome-seq data are not causally related to clinical outcome.

To show the confounding effects of copy number variation on allele frequency distributions, we relied on a crude measure of genome-wide copy number variations (i.e. CNV standard deviation across SNVs), and showed that it is correlated with MATH and is predictive of patient survival in some cases. However, more in-depth analysis of copy number variations is required to fully explore the clinical relevance of CNV burden in tumors. Nevertheless, our results are consistent with copy number aberrations being a more important predictor of outcome, which has previously been reported over pooled TCGA cancer types^3^. In our analysis most tumor types showed a regular, though not always statistically significant, trend between increased copy number variation and shorter survival time. Notably, high copy number variation has been previously reported to be associated with poor patient survival in serious endometrial carcinoma^39^. To determine the association between clinical features and CNV standard deviation, we also separately applied clinical feature selection (see *Methods*) to UCEC, and found that histologic diagnosis is the strongest predictor, with increased CNV variation for serous carcinoma (Fig. S15).

Ovarian cancer is opposite to the common copy number trend, in that tumors with more CNV variation are associated with longer survival. However, in ovarian cancer BRCA1 deletions often lead to the high-CNV tandem duplicator phenotype, and this phenotype has been found to have better clinical outcome^40^. Indeed, in the TCGA data we observe that increased deletions in BRCA1 are correlated with CNV standard deviation (Fig. S16), suggesting that the ovarian cancer effect is mediated by the tandem duplicator phenotype.

Employing the full spectrum of allele frequency distributions in clinical studies would require multidimensional survival analysis methods, which are sensitive to regression assumptions, or alternatively increasing statistical power by use of larger cohorts. Due to these limitations, in this paper we only focused on reduction of the distribution to a single quantity, namely MATH. Our analysis indicates that allele frequency distributions as quantified by MATH score are not useful clinical prognostic measures across cancer types. However, our work does not rule out the possibility of defining a set of clinically meaningful measures derived from allele frequency distributions. The issue however is that the space of possible measures is dramatically large, and, unless a more mechanism-driven approach is adopted, one would easily encounter underpowered statistics. To reflect this point we explored 11 extra statistics (Fig. S17) and found very little association to patient survival. The only exception was COAD when it was called by SomaticSniper.

A more direct measure of tumor heterogeneity is the cancer cell fraction (CCF), which is the fraction of cells having a specific SNV. We determined the CCFs for all cancers and mutation callers using PyClone^7^ (see Methods), and calculated MATH score using CCFs instead of allele frequencies (AF). We found that CCF MATH and AF MATH are positively correlated (Fig. S18, mean Pearson correlation coefficient 0.55, with minimum 0.30 for HNSC-SomaticSniper, and maximum 0.82 for GBM-MuTect). Similar to AF MATH, higher CCF MATH was associated to poorer patient survival in UCEC-MuTect, however this result was not robust across mutation callers (Fig. S18).

In addition, we explored the importance of tumor purity on our results. Theoretically, tumor purity scales all mutant allele frequencies by a constant and should not affect MATH score. Order statistics such as KS test would not be affected by purity for a similar reason. However, dependence of mutation-calling errors on allele frequencies, and the removal of allele frequencies smaller than 0.1 in our pipeline are sensitive to purity scaling. To directly assess the effect of tumor purity we therefore compared MATH score against purity scores provided by TCGA, and found very little association between the two (−0.1 < *r* < 0.1, Fig. S19). Next, we removed samples with purity below 0.8 and compared allele frequency distributions across mutation callers. The results were similar to what we have described in Fig. 2. In the purity filtered datasets, survival analysis of MATH score did not lead to significant results for any cohort. As in the unfiltered analysis, CNV standard deviation was associated with patient survival in UCEC-MuTect and OV-SomaticSniper (Fig. S19).

Overall, we conclude that heterogeneity as quantified by MATH is not a strong predictor of patient survival, whether it is applied to allele frequencies or CCFs. CCF inference is prone to several errors, which can arise from many factors including strong priors in the inference algorithms, errors in allele frequency and copy number measurements, gross underestimation of SNV counts private to small subclones, and subsampling of the tumors. Due to these issues CCF distributions may have unresolved prognostic powers which would require deeper genetic sequencing, use of more informative measures, and an increase in the number of patients and clinical data.

Despite these findings, we cannot conclude that subclonal heterogeneity is irrelevant to survival, as the processes by which resistant populations develop remain poorly understood even though they are crucial to outcome. For example, our work shows that gross estimates of subclonal heterogeneity from exome-seq data have little predictive power, but other studies have shown that resistance can arise from populations too small to be detectable by exome-seq^41^. Higher resolution measurements of subclonal heterogeneity may solve this challenge, and further development of robust computational analyses will be a critical part of such measurements.

Finally, we have shown that multiple evolutionary histories can lead to the same allele frequency distributions, and that different allele frequency distributions can have the same MATH score. This degeneracy of scenarios leading to the same measurement is an important reason why new descriptors of heterogeneity are needed. Future surveys over different evolutionary scenarios should be valuable for distinguishing what types of heterogeneity measurements are most likely to reveal features predictive of survival.

## Conclusions

We studied the robustness of allele frequency distributions to mutation calling procedures across TCGA cancers and explored their prognostic power for patient survival. We found that mutation callers differ significantly in their estimates of the distributions of allele frequencies for individual tumors, and that the association between allele frequency distribution and survival is non-robust to these differences. The major exception is uterine corpus endometrial carcinoma, but the observed effect is mediated by copy number variation rather than subclonal heterogeneity. Our work has implications for cancer precision medicine, as we show that current measures of heterogeneity are not predictive of survival except through an indirect association with copy number variation.

## Methods

### Computational Time

The average time required to run a task was 2 hours and 25 minutes. We ran a total of 14865 jobs, with total wall time of 1496 days.

### Sample Selection

Samples were chosen only if tumor/normal Illumina exome-seq data existed with alignment to hg19, and corresponding clinical and Affymetrix SNP Array somatic copy number variation data were present. SNVs were called by three mutation callers and samples were discarded if any mutation caller did not find any somatic SNVs in that sample. If multiple normal samples were available, blood has been preferred over solid tissue. For tumors, primary solid tumor was used if available. The main anomaly was in SKCM, where most TCGA samples are metastatic (Fig. S2).

### Mutation Caller Parameters

Mutation calling was done with minor tweaks to the default parameters of each mutation caller (Table 1). For MuTect, these flags control proximal gaps, poor mapping quality, strand bias, clustered positions, mutations in normal sample, low tumor/normal mutation likelihood ratio, and presence in dbsnp. For SomaticSniper we set parameters to ignore loss of heterozygosity and gain of reference. Furthermore SomaticSniper uses a Phred-scaled value, ‘somatic score’, to determine somatic mutations. We have used a high-confidence threshold for this value to improve precision of the algorithm (−Q 40). The parameters for VarScan set minimum for coverage and allele frequency to limit the number of reported mutations. However these parameters will be overridden in the post-processing step.

**Table 1:** Mutation caller parameters.

Caller	Parameters
MuTect 1.1.7	default (tumor_1od = 6.3 fraction_contamination = 0.02 normal_1od = 2.2 strand_artifact_lod = 2.0 strand_artifact_power_threshold = 0.9 dbsnp_normal_1od = 5.5 gap_events_threshold = 3 fraction_mapq_threshold = 0.5 pir_median_threshold = 10.0 pir_mad_threshold = 3.0 required_maximum_alt_allele_mapping_quality_score = 20 max_alt_alleles_in_normal_count = 2 max_alt_allele_in_normal_fraction = 0.03)
SomaticSniper 1.0.5.0	-Q 40 -L -G
VarScan 2.3.9	somatic –min-coverage 10 –min-coverage-normal 10 –min-coverage-tumor 10 –min-var-freq 0.05

### Postprocessing Filters

We applied post-processing filters on the output VCF files to extract the somatic SNVs. For MuTect and VarScan we selected SNVs with FILTER = PASS, and in all cases we only used SNVs which had zero allele frequency for normal sample and a non-zero value for the tumor. A read depth filter of minimum 50 was also applied to both normal and tumor samples. Furthermore, we discarded SNVs which had tumor or normal allele frequency $$ < 0.1$$. For more detail on mutation calling process and parameters see Fig. S1 and Table 1.

### Statistical Distances

To calculate the earth mover’s distance (EMD)^42^, we produced histograms of allele frequencies for each sample using bins of size $0.025$ and followed the procedure in^43^ for chain-connected spaces. For two probability distributions ${f}_{1}(x)$ and ${f}_{2}(x)$ with respective normalized histogram abundances ${f}_{\mathrm{1,}j}$ and ${f}_{\mathrm{2,}j}$ at *j*’th bin (with $j\le N$), EMD can be written as^43^:

2 $${\rm{EMD}}({f}_{1},{f}_{2})=\sum _{i=1}^{N-1}\sum _{j=1}^{i}|\,{f}_{\mathrm{1,}j}-{f}_{\mathrm{2,}j}|$$

Quantities reported in this paper are the median of this value across each cohort (Fig. 2C).

We also used another method for quantifying differences between distributions of allele frequencies, where we smoothed the histogram of allele frequencies using Gaussian kernel density estimation with standard deviation $\sigma =0.02$, leading to two continuous functions ${f}_{1}(x)$ and ${f}_{2}(x)$. We then calculated the cumulative absolute difference of these two functions using ${\int }_{0}^{1}|\,{f}_{1}(x)-{f}_{2}(x)|dx$. Again the median of cohort was calculated as a single measure of statistical distance (Fig. S5).

Finally, we also used a non-parametric statistical distance between allele frequency distributions which relies on the average ${L}^{2}$ distance between two distribution functions^35^. The statistic is defined as ${\int }_{0}^{1}{[{f}_{1}(x)-{f}_{2}(x)]}^{2}dx$, where ${f}_{1}(x)$ and ${f}_{2}(x)$ are the Gaussian kernel density estimations of the two functions (bandwidth = 0.05). We calculated the corresponding p-values by applying a permutation test to each pair of allele frequency sets for which the test statistic was being calculated. This procedure was repeated 100 times and the ratio of tests which were more extreme than the non-permuted test was reported as the p-value.

### Allele Frequency and Read Depth

Read depth and allele frequency were extracted from the FORMAT field of VCF files. Multiallelic and biallelic sites were treated similarly and their allele frequencies corresponded to the ratio of alternative alleles to reference alleles. For MuTect and VarScan the allele frequency computed by the software was directly extracted from VCF files, and for SomaticSniper this was done by using the reference and alternative read counts reported by the software.

### Copy Number Data

For copy number analysis we used masked copy number level 3 TCGA data measured by Affymetrix SNP Array 6.0 which is readily aligned to hg19 and normalized for somatic copy number detection by removal of frequent germline copy number variations. The quantity used throughout the paper is the *Segment Mean* column in the ‘nocnv’ files which is either used directly or determined at the SNV locus of interest. This quantity is the average log2 ratio of copy number of the tumor compared to reference (=2) within a segment of the genome where internal changes are minimal. Positive values correspond to amplification and negative values correspond to deletion. Wherever mentioned in the text, copy number filtering was done according to^44^ where loci with $|CNV| > 0.2$ were removed.

### Survival Data

Survival information was gathered from the TCGA data portal by parsing the clinical json files for field *diagnoses*.*days_to_death* as survival time. In cases where this field was not available, we used *diagnoses*.*days_to_last_follow_up* instead. The field *diagnoses*.*vital_status* was used for determining censored data.

### Survival Analysis

Survival analysis was done using Kaplan-Meier curves and log rank test method from the Lifelines Python package^45^. Only the first 4 years of survival information was used for analyses and any sample with longer overall survival was censored at this time point.

Survival difference values (Fig. S10) were defined by calculating the Kaplan-Meier curves (Figs S8, S11) for two groups with high and low variability score (divided across the median of the score used). We then integrated the absolute value of difference of survival functions:

3 $$\frac{1}{{T}_{max}}{\int }_{0}^{{T}_{max}}[{S}_{High}(t)-{S}_{Low}(t)]dt$$

where ${S}_{High}(t)$ and ${S}_{Low}(t)$ are the survival functions of the two groups and ${T}_{max}$ corresponds to the maximum time of data collection (4 years). This definition leads to a quantity between $-\,1$ and $1$ which is more negative if the high score group has worse survival, and more positive if the high score group has better survival.

### Expression Analysis

To determine how copy number amplification influences expression, we downloaded the corresponding RNAseq expression results (RPKM values) of UCEC for each sample from the TCGA database.

For gene $g$ in patient $p$, let matrices ${r}_{p,g}$, ${c}_{p,g}$, and ${s}_{p,g}$ respectively be the RPKM value, CNV value, and SNV status ($1$ if mutated, $0$ otherwise) of the gene. Furthermore, for total of $N$ patients let us define ${\mathscr{A}}({x}_{p,g})$ as the average of positive elements of matrix ${x}_{p,g}$ along the columns:

4 $${\mathscr{A}}({x}_{p,g})\equiv \frac{\sum _{p=1}^{N}{x}_{p,g}}{\sum _{p=1}^{N}u({x}_{p,g})}$$

where $$u(x)=\{\begin{array}{c}0,\,x\le 0,\\ 1,\,x > 0.\end{array}$$ is a step function that includes the zero coordinate. The reason for this definition of ${\mathscr{A}}({x}_{p,g})$, is to calculate few different versions of gene expression averages across cohorts based on different criteria illustrated below:

1. mean RPKM (amplified, with SNV):
  5 $${\mathscr{A}}({r}_{p,g}\times u({c}_{p,g}-{c}_{2}^{th})\times {s}_{p,g})$$
2. mean RPKM (not amplified, without SNV):
  6 $${\mathscr{A}}({r}_{p,g}\times u({c}_{1}^{th}-{c}_{p,g})\times \mathrm{(1}-{s}_{p,g}))$$
3. mean RPKM (amplified, without SNV):

7 $${\mathscr{A}}({r}_{p,g}\times u({c}_{p,g}-{c}_{2}^{th})\times \mathrm{(1}-{s}_{p,g}))$$

Here $\times$ is ordinary element-wise product. ${c}_{1}^{th}=0.1$ and ${c}_{2}^{th}=1.0$ are the CNV thresholds for no amplification and amplification respectively. Note that since amplified SNVs are rare, the averaging in equation (5) could be dropped, rendering this quantity effectively as the single gene RPKM. Furthermore, in cases that the denominator of equation (4) is zero (i.e. no sample meets the criteria), the gene is discarded. Associations between these expression values are reported in Fig. S14.

To determine the effects of tumor suppressors and oncogenes on expression results, we downloaded the list of tumor-suppressor genes from TSGene^46,47^ and oncogenes from ONGene^48^ and removed the genes existing in either of the lists.

### CNV Standard Deviations

Throughout the text CNV standard deviation (std) of a sample is calculated as the standard deviation of CNVs at all loci that have an SNV.

On the other hand, whole genome (WG) CNV standard deviation is calculated independent of SNVs, by uniform sampling of the genomic regions for which copy number information is available. Equivalently, we can write:

8 $${\rm{WG}}\,{\rm{CNV}}\,{\rm{std}}=\sqrt{\sum _{i=1}^{N}{p}_{i}{({c}_{i}-\overline{c})}^{2}}$$

where $\bar{c}=\sum _{i=1}^{N}{p}_{i}{c}_{i}$ is the mean CNV across the genome, ${c}_{i}$ is the CNV of copy number segment *i*, *N* is the total number of segments, and ${p}_{i}={l}_{i}/{\sum }_{j=1}^{N}{l}_{j}$ is the fraction of genome covered by segment *i* with genomic length *l*_*i*_.

### Mathematical Modeling

The mathematical model presented in this paper is a linear of model of tumor evolution^36^, where any driver mutation leads to a selective sweep and the remaining mutations are passenger mutations. All the mutations before the last selective sweep are clonal with allele frequency 0.5. The remaining mutations follow the neutral model, with cumulative distribution of allele frequency $f$ proportional to $\mathrm{1/}f$^24^. Then, the overall allele frequency distribution is the combination of these two distributions sampled according to their occurrence rates (see Supplementary Material):

9 $$P(F\le f)=\{\begin{array}{cc}0, & f < {f}_{min},\\ \frac{\nu }{2}(\frac{1}{{f}_{min}}-\frac{1}{f}), & {f}_{min}\le f < \,0.5,\\ 1, & f=\mathrm{0.5.}\end{array}$$

where $\nu =\frac{2w{f}_{min}}{1-2{f}_{min}}$, $w$ is the ‘neutral fraction’ and is defined as the fraction of all mutations that are neutral, and ${f}_{min}$ is the minimum measurable allele frequency. The latter quantity is necessary in order to avoid singularity at $f=0$. In our simulations we set ${f}_{min}=0.1$. The results of simulations with no measurement noise (Fig. 5C) were calculated using 300 samples from this distribution, and their variations reflect this sampling error.

We derived a closed form for MATH score of the model as a function of $w$ (see Supplementary Material for derivation):

10 $${\rm{M}}{\rm{A}}{\rm{T}}{\rm{H}}=148.26\times \{\begin{array}{cc}0, & w\le \frac{1}{2},\\ \frac{1}{2\varphi }-1, & \frac{1}{2} < w\le \frac{1}{2}+{\rm{\Delta }}w,\\ \frac{\sqrt{{\nu }^{2}+{\varphi }^{2}}-\nu }{\varphi }, & w > \frac{1}{2}+{\rm{\Delta }}w.\end{array}$$

where $${\varphi }=\frac{\nu {f}_{min}}{\nu -{f}_{min}}$$, and $${\rm{\Delta }}w=\frac{\sqrt{1+32{f}_{min}^{2}}-1}{16{f}_{min}}$$.

The allele frequency measurement error can be modeled by a binomial distribution, where observing a mutation on a sequencing read is a Bernoulli trial. For TCGA whole exome sequencing, read depths are quite large (on average 100×) and this error can be approximated by a Gaussian distribution. Therefore, to implement allele frequency noise we drew 300 samples from the distribution in equation (9) and used each resulting value $f$ to sample from the normal distribution ${\mathscr{N}}(f,\frac{f\mathrm{(1}-f)}{N})$. Here $N=100$ is the sequencing read depth.

### Cancer Cell Fractions

To determine cancer cell fractions (CCF), we used PyClone^7^ in the allele-agnostic mode by setting the flag ‘–prior’ to ‘total_copy_number’. For input tsv files, we set normal_cn = 2, minor_cn = 0, and major_cn = round $\mathrm{(2}\times {2}^{CNV})$, where in the latter equation, CNV is the log2 ratio segmentation information from TCGA SNP Array data. In a few samples a small number of loci had major_cn = 0. We removed these loci from our analysis. Furthermore, to speed up PyClone runtime, if a sample had more than 500 SNVs we randomly removed excess loci.

### Alignment to GRCh38

Genomic alignments can lead to different SNVs and/or allele frequencies, depending on read quality and genomic repeats. As a confirmatory step we explored this effect by comparing our results for selected samples against alignments to the new human genome (hg38). To achieve this, we downloaded 227 TCGA breast cancer BAM files of whole exome sequencing data from Cancer Genomics Hub (CGHub)^49^. After converting the BAM files to FASTQ files, we remapped the FASTQ files to hg38 reference genome with the Burrows-Wheeler Aligner (BWA)^50^. The somatic SNVs were called by MuTect^28^. We found that the allele distributions contained less SNVs overall, but their shapes were relatively similar (Fig. S20).

### Clinical Feature Selection

Random forest feature selection of clinical data (Fig. S15) was done using *ExtraTreesClassifier* function from Python package scikit-learn^51^, with default parameters and 250 estimators. Features with $\mathrm{20 \% }$ or more unknown values were discarded and the remaining unknown values were set to the median of the feature for numerical features or the mode for categorical features. Data labeling was done by comparing CNV standard deviation to its median.

## Electronic supplementary material

Supplementary Information
