# Supplementary material for: Distribution-based measures of tumor heterogeneity are sensitive to mutation calling and lack strong clinical predictive power
Source: Sci Rep. 2018 Jul 30;8:11445. doi: 10.1038/s41598-018-29154-7 (PMC6065409; doi:10.1038/s41598-018-29154-7)
Supplement: Supplementary file 1 — Supplementary Information [file 41598_2018_29154_MOESM1_ESM.pdf]

Distribution-based measures of tumor heterogeneity are sensitive to mutation calling and lack strong clinical predictive power

Javad Noorbakhsh,[1] Hyunsoo Kim,[1] Sandeep Namburi,[1] and Jeffrey Chuang[1,2,*]

[1] *The Jackson Laboratory for Genomic Medicine, Farmington, CT, USA*

[2] *University of Connecticut Health Center, Department of Genetics and Genome Sciences, Farmington, CT, USA*

[*] jeff.chuang@jax.org

SUPPLEMENTARY MATERIAL

Figures

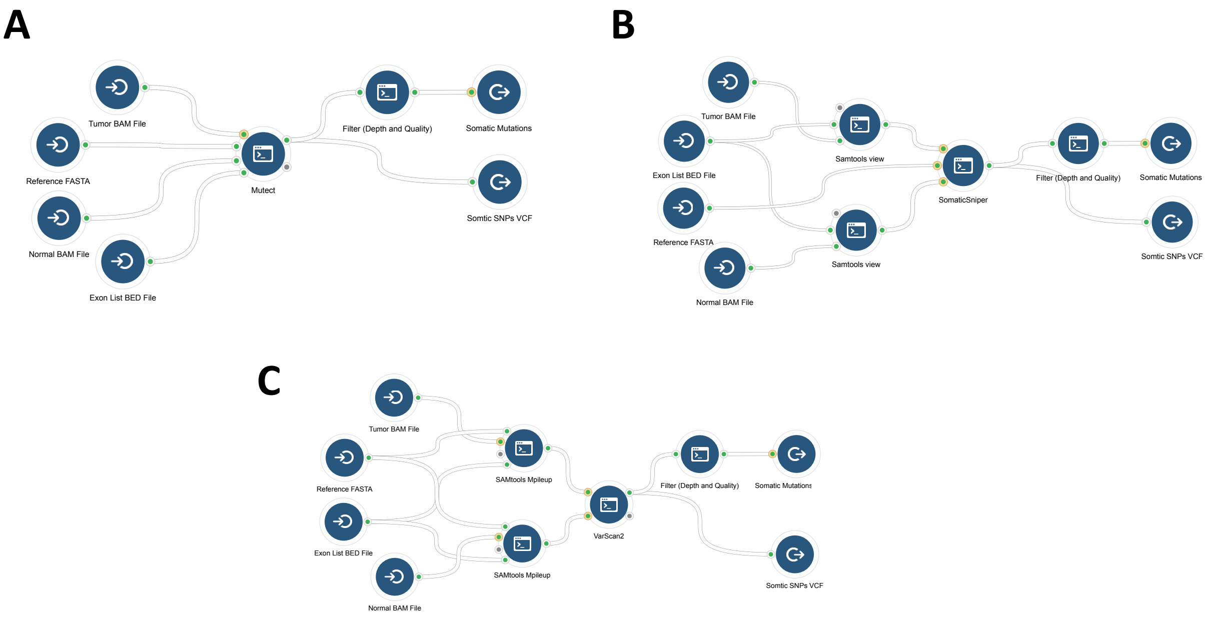

FIG. S1. **Supplementary Information.** Mutation calling pipelines on CGC for A) MuTect, B) SomaticSniper, and C) VarScan

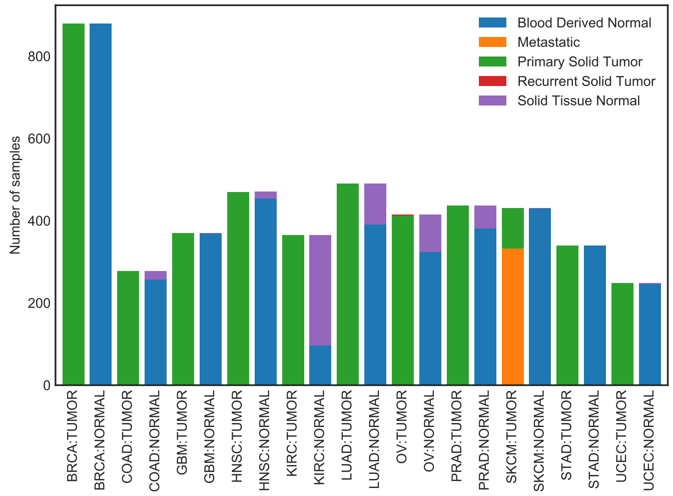

FIG. S2. **Supplementary Information.** Distribution of sample types used for each cancer type.

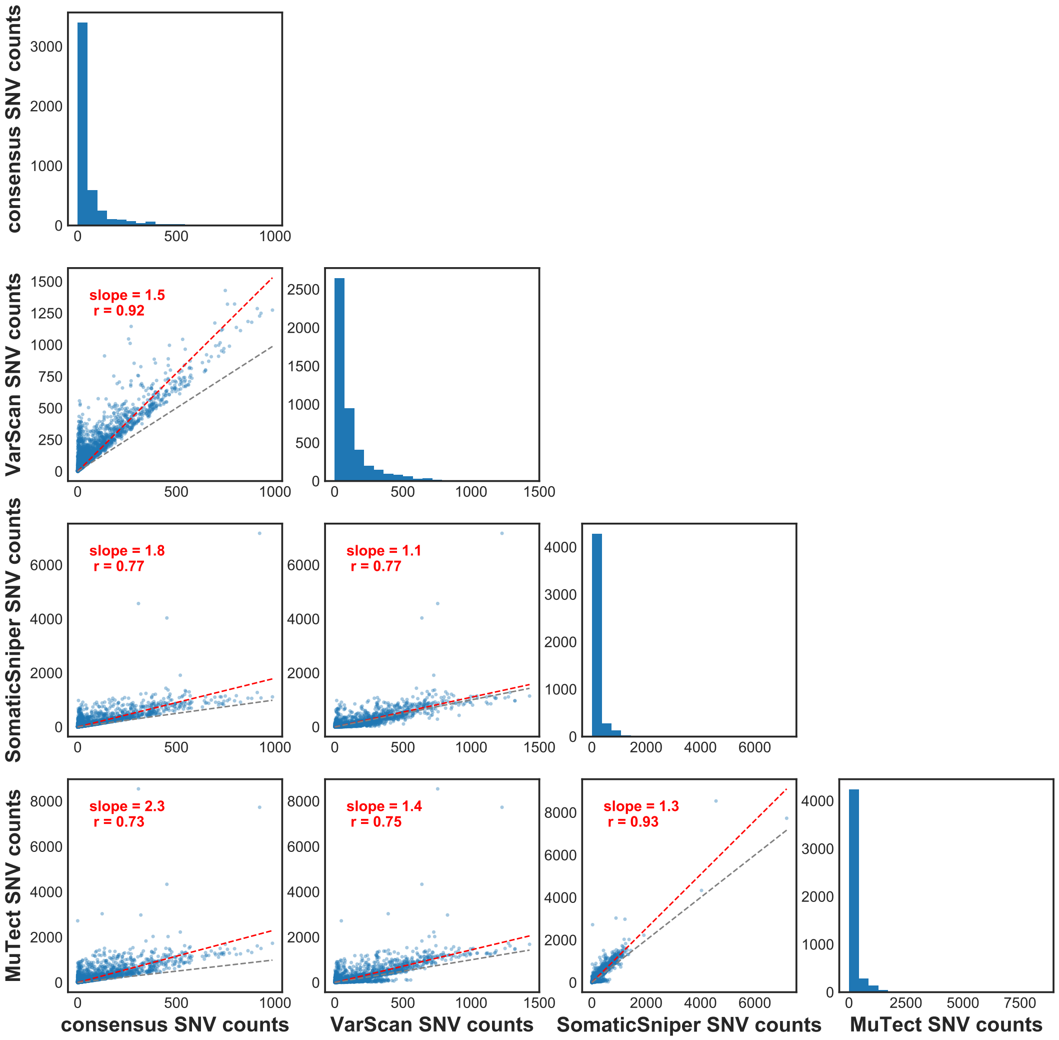

FIG. S3. **Supplementary Information.** Pan-cancer comparison of SNV counts per sample called by different mutation callers and consensus SNVs along all callers. Each dot is an individual sample. Red line is the best fit line with zero intercept (slope and correlation coefficient included on the plots). Gray line is the line with slope one.

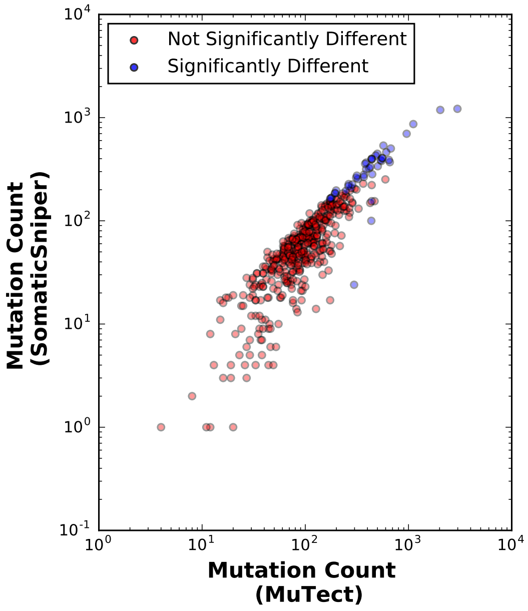

FIG. S4. **Supplementary Information.** SNV counts for different samples within HNSC compared between two mutation callers. Samples which have distributions that are significantly different according to the Kolmogorov-Smirnov test are shown by blue dots.

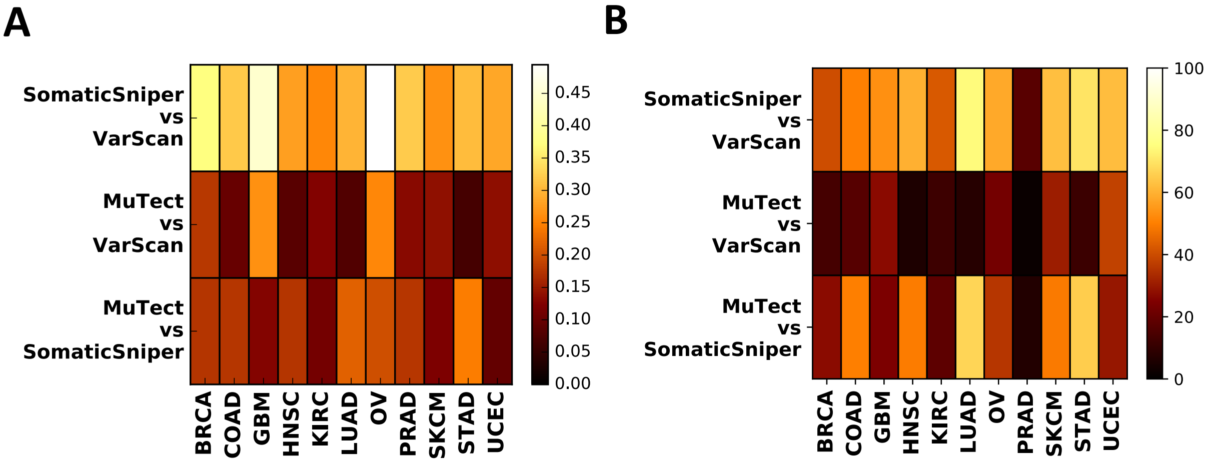

FIG. S5. **Supplementary Information.** Comparison of allele frequency distributions using A) cumulative absolute difference of smoothed histograms and B) Distribution of samples which significantly differed based on the quadratic statistics proposed by Qi Li [1] (Bonferroni corrected with significance threshold $\alpha = 0.05$). The percentage of samples that fall below this threshold are on average $38.3 \pm 18.4\%$ for MuTect vs SomaticSniper, $17.1 \pm 10.7\%$ for MuTect vs VarScan, and $53.6 \pm 15.1\%$ for SomaticSniper vs VarScan comparisons.

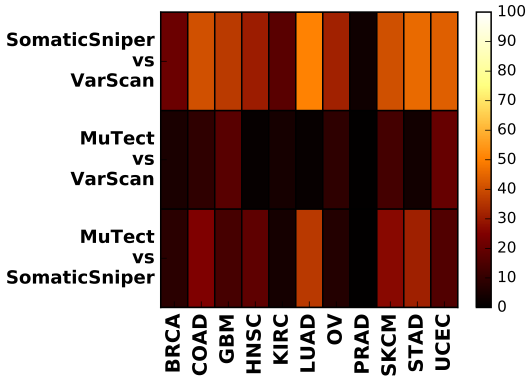

FIG. S6. **Supplementary Information.** Comparisons of allele frequency distributions for different mutation callers. Percentage of significantly different samples (as determined by Kolmogorov-Smirnov test with $p < 0.05$) for mutation caller pairs shown for all cancers with copy number filtering : $|CNV| < 0.2$

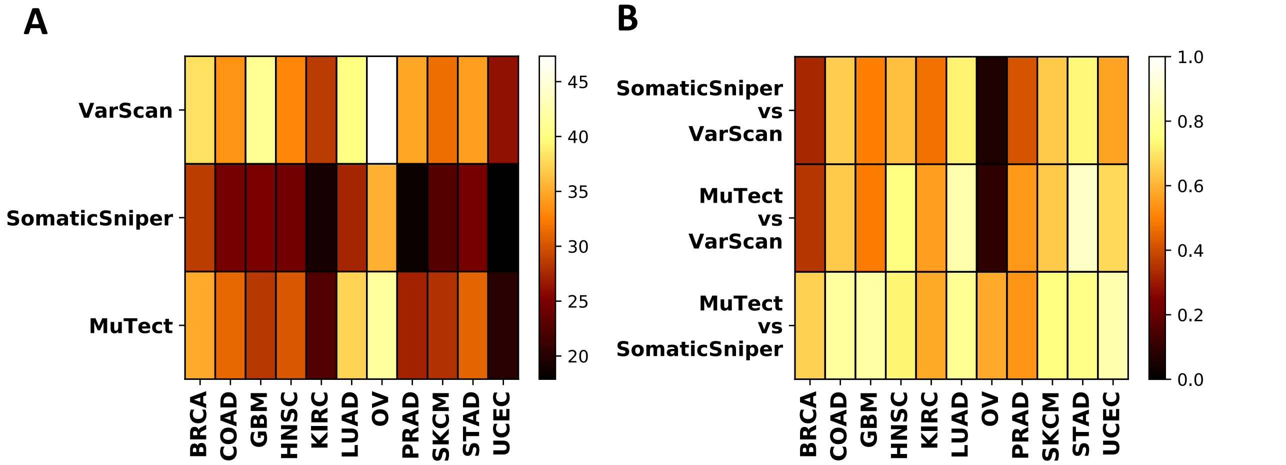

FIG. S7. **Supplementary Information.** A) Median of MATH score for all cancers and mutation callers. B) Spearman correlation coefficient of MATH scores for each cancer type called by pairs of different mutation callers.

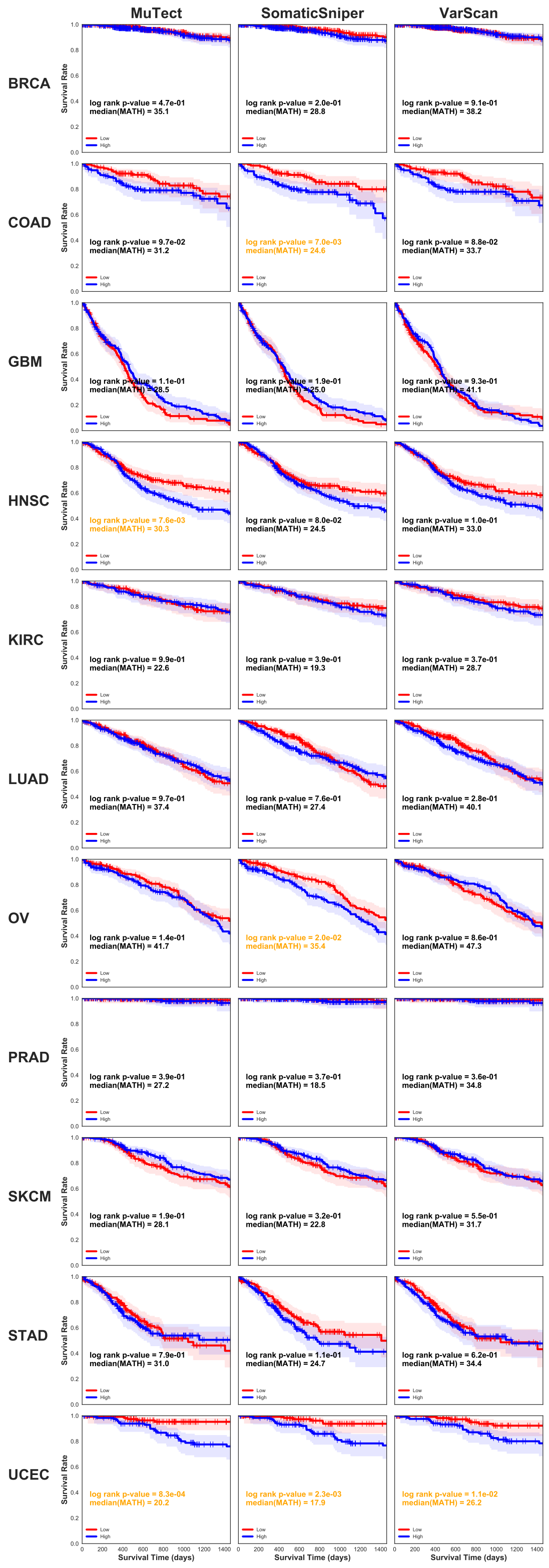

FIG. S8. **Supplementary Information.** Survival curves for all cancers and mutation callers using median MATH score as separator. The values for median MATH score and the log rank test p-values are included on each plot. p-values smaller than 0.05 are marked in orange.

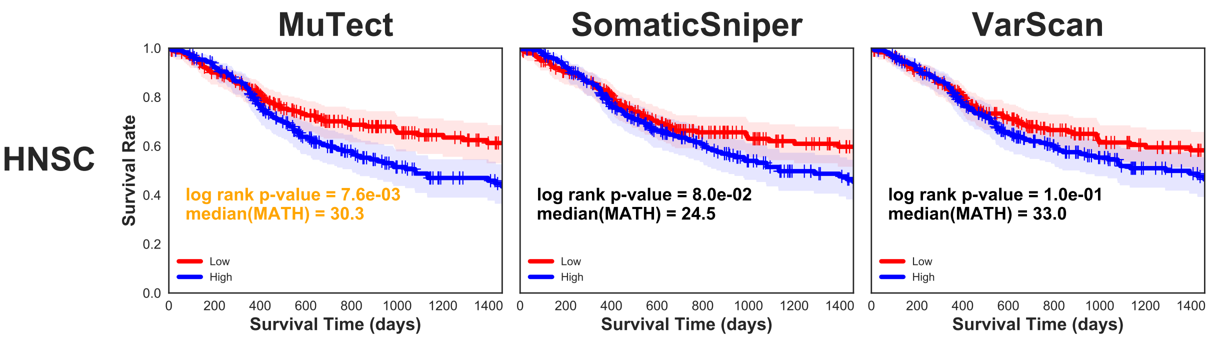

FIG. S9. **Supplementary Information.** Survival analysis using MATH score of the subset of HSNC samples used in [2]

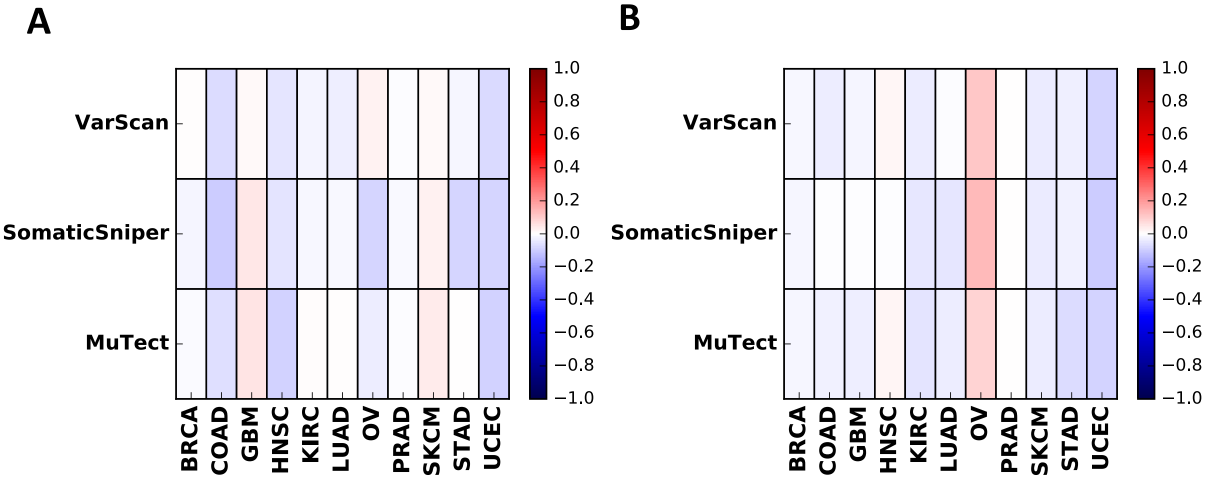

FIG. S10. **Supplementary Information.** A) Survival curve difference for groups separated by MATH score, B) survival curve difference for groups separated by CNV standard deviation

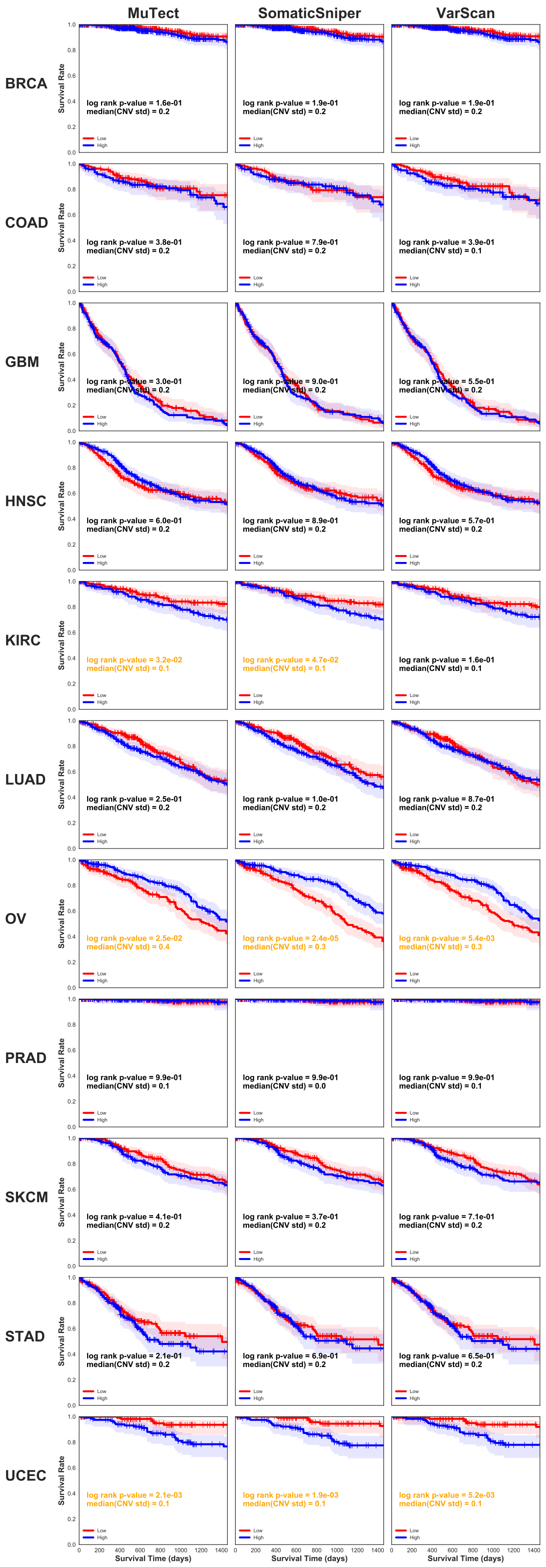

FIG. S11. **Supplementary Information.** Survival curves for all cancers and mutation callers using median of CNV std as separator. The values for median of CNV std and the log rank test p-values are included on each plot. p-values smaller than 0.05 are marked in orange.

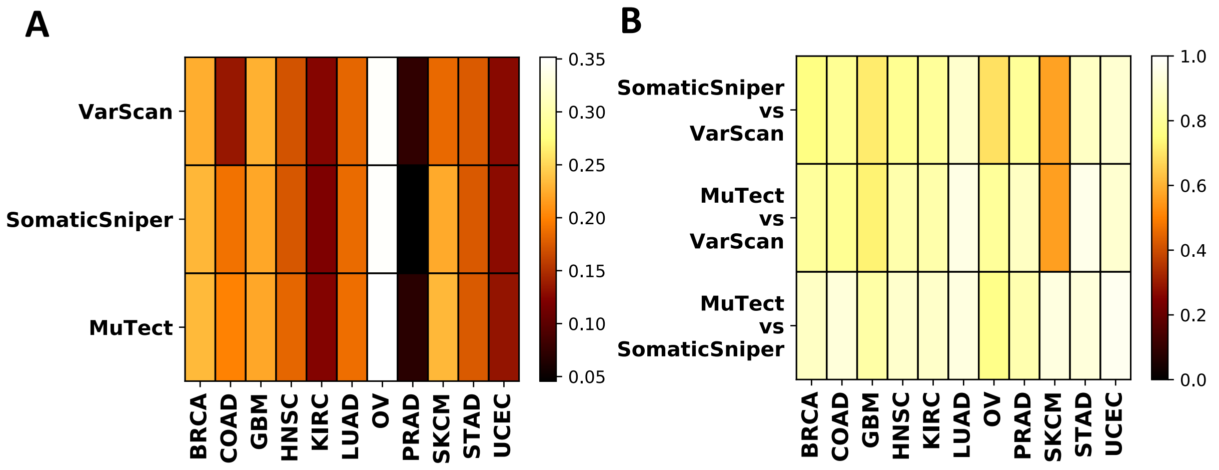

FIG. S12. **Supplementary Information.** A) Median of CNV std score for all cancers and mutation callers. B) Spearman correlation coefficient of CNV std scores for each cancer type called by pairs of different mutation callers.

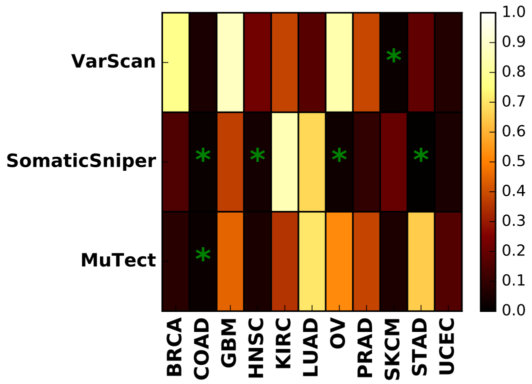

FIG. S13. **Supplementary Information.** Log rank test p-values for comparison of low versus high MATH score when filtered by copy number ($|CNV| < 0.2$). Stars represent significant results as determined by $\alpha < 0.05$

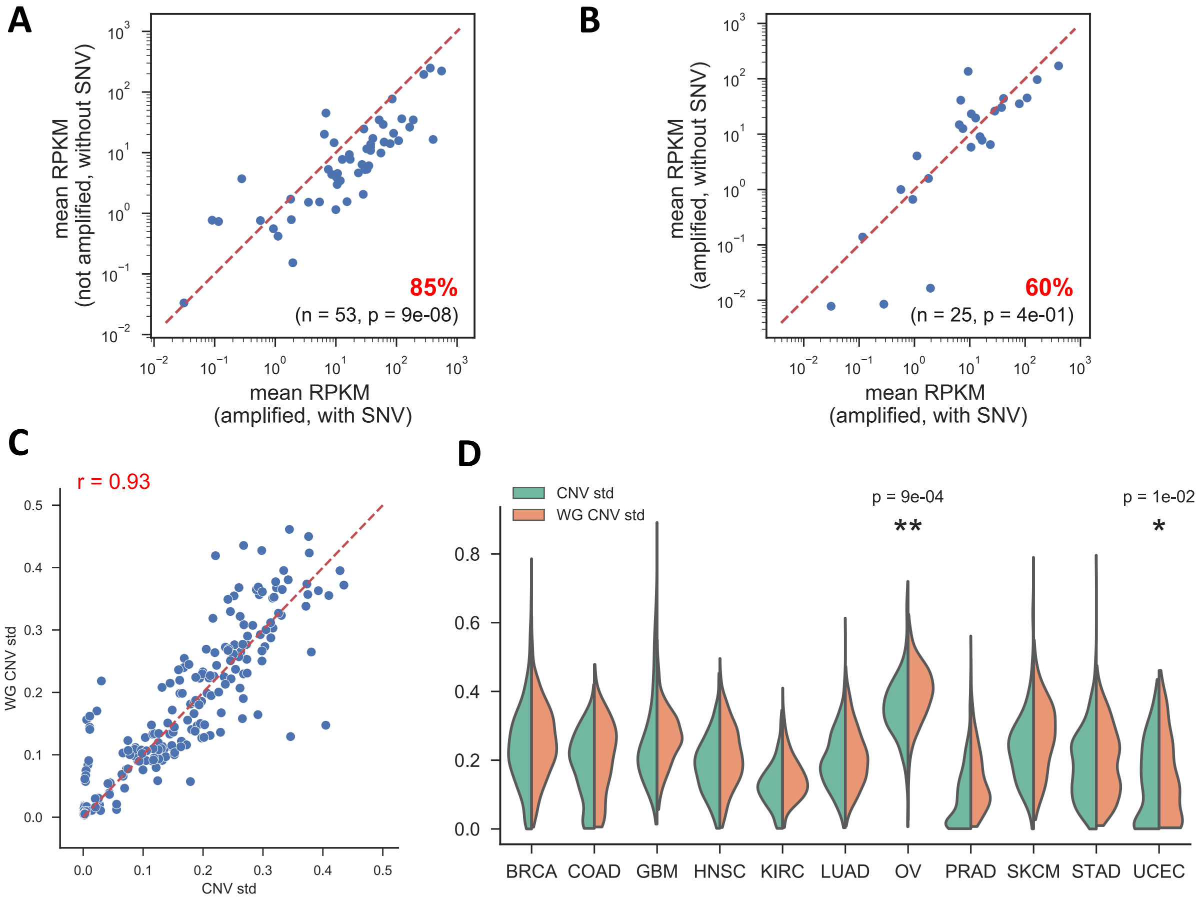

FIG. S14. **Supplementary Information.** Analysis of SNV/CNV interactions. A) Plot of effect of SNVs and CNVs on gene expression in UCEC. Each data point is a single gene for which average expression across samples without SNV and without copy number amplification is plotted against average expression across samples with SNV and with copy number amplification (see *Methods* for detailed definition). Dashed red line is the identity line and the percentage of data points falling below this line is shown in red. n is the number of genes studied, and p-value is calculated using Wilcoxon signed-rank test. B) Similar plot to panel (A) for average expression across samples without SNV, and with copy number amplification against average expression across samples with SNV and with copy number amplification. C) Plot of WG CNV std against CNV std (see *Methods*). Each dot is a single UCEC sample. Red dashed line is identity line, and r is the Spearman correlation coefficient. D) Distribution of WG CNV std compared to CNV std, and its clinical associations. p-values correspond to log-rank test for samples split across median of WG CNV stds (only significant values shown). *: $p < 0.05$, **: $q < 0.05$ (Benjamini-Hochberg correction). All SNVs called by MuTect.

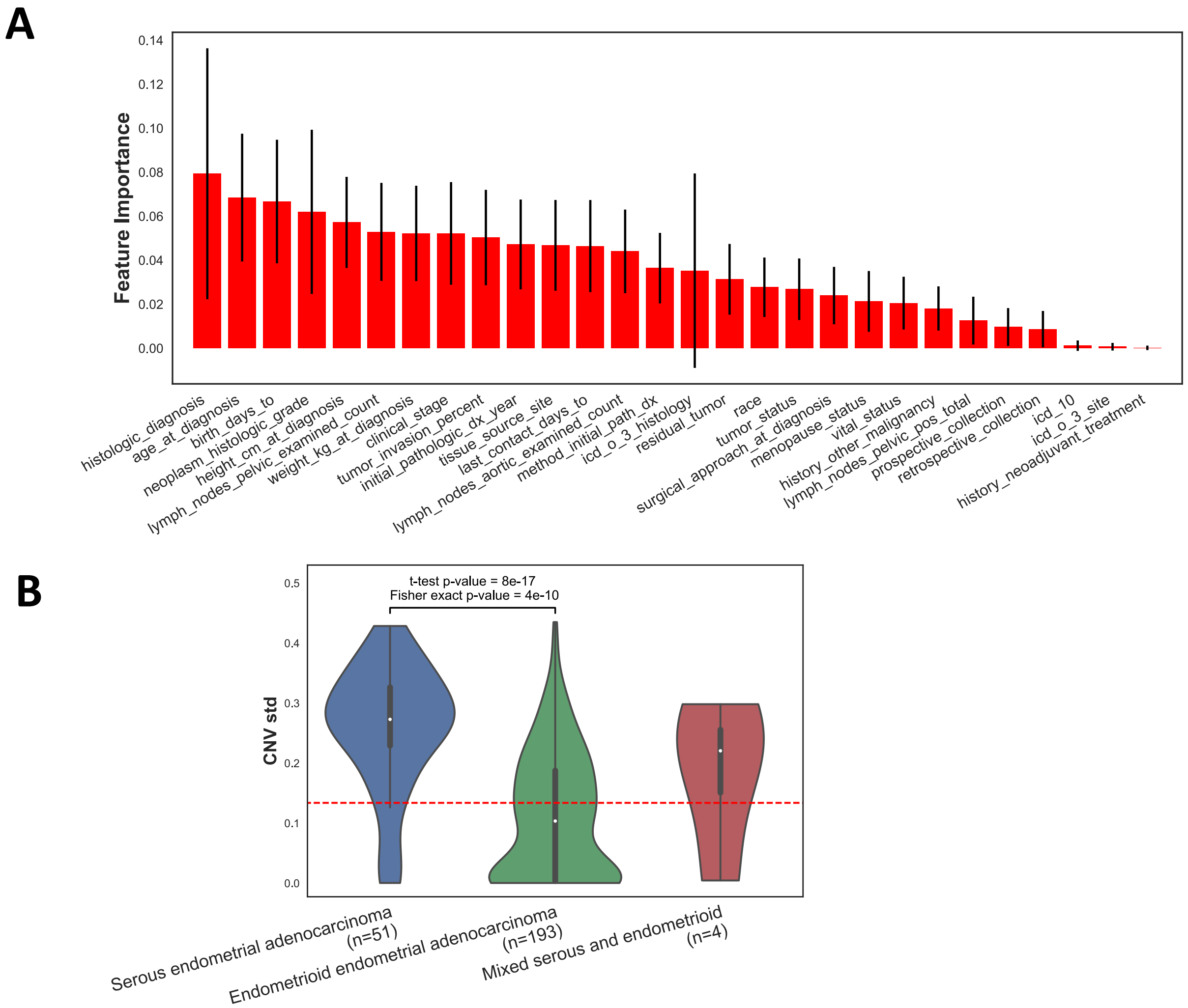

FIG. S15. **Supplementary Information.** A) Clinical data sorted according to their importance in classifying high and low CNV std groups (divided across median) of UCEC. Results were achieved using random forest feature selection. B) Comparison of CNV standard deviation for the three histologic subtypes of UCEC. Dashed red line corresponds to median of CNV standard deviation. The Fisher's exact test is calculated in comparison to this line.

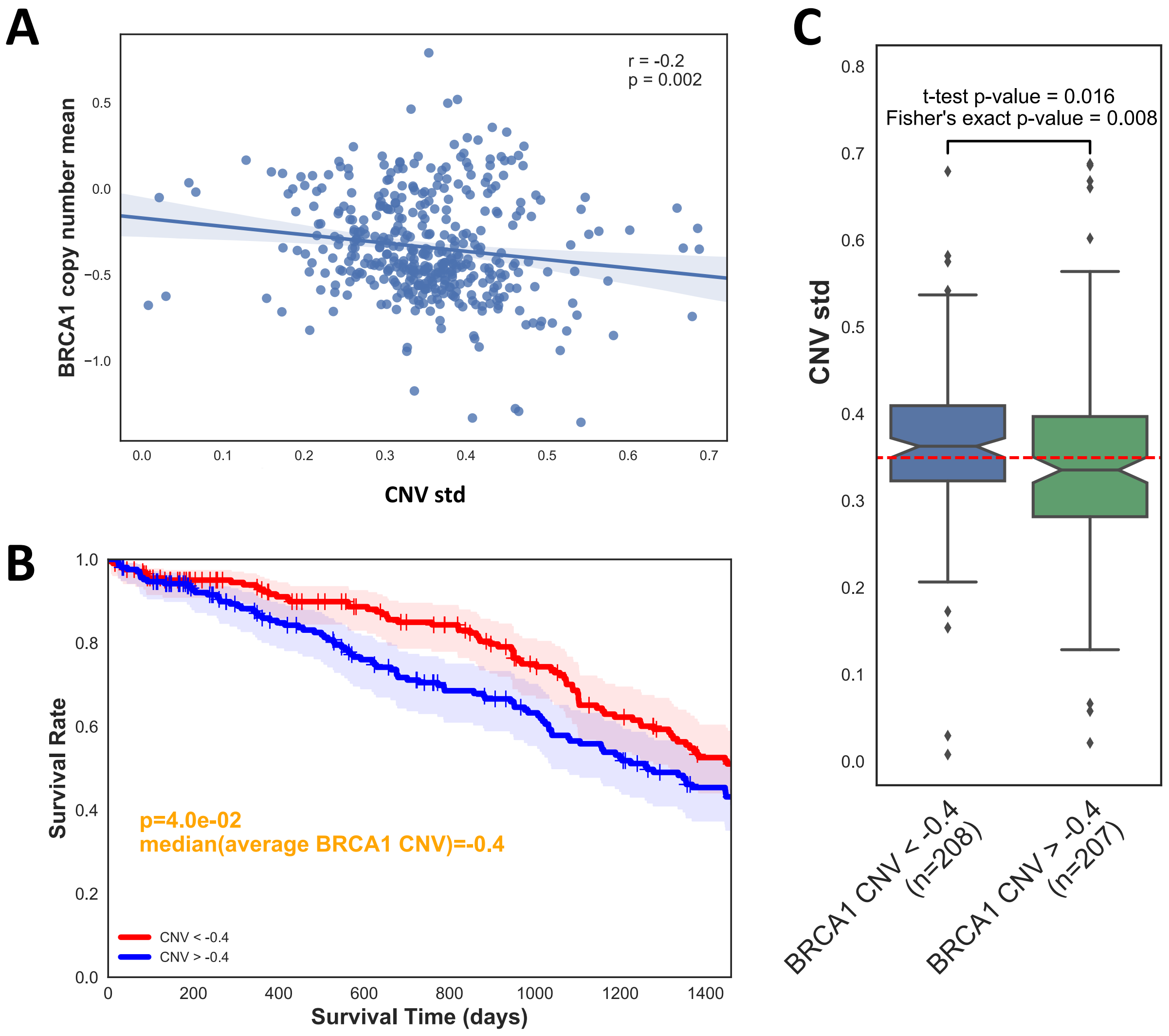

FIG. S16. **Supplementary Information.** Comparison of exome-wide copy number standard deviation (at loci called by SomaticSniper) against BRCA1 copy number for OV. A) Average copy number across BRCA1 plotted against copy number standard deviation showing a negative correlation. Each dot corresponds to one patient. B) Survival analysis of patients divided in relation to median value of BRCA1 copy number average C) Comparison of CNV standard deviation of the two groups in B. Dashed red line corresponds to median of CNV standard deviation. Fisher's exact test is calculated in comparison to this line.

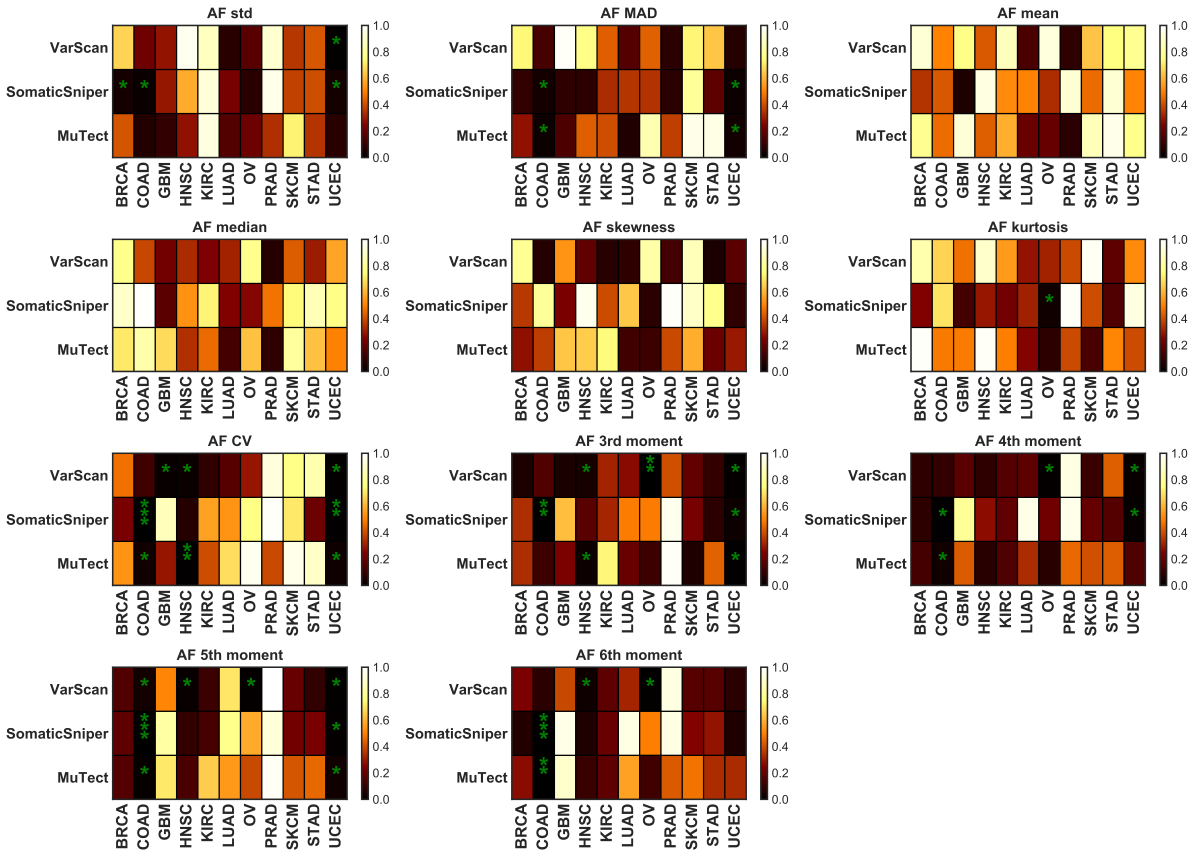

FIG. S17. **Supplementary Information.** Survival analysis log rank test p-values for 11 different statistics derived from allele frequency distributions. Stars corresponds to values smaller than significance threshold 0.05. Double stars show significant results after Benjamini-Hochberg correction across all cancer types and mutation callers within individual plots. Triple stars correspond to Benjamini-Hochberg correction across all 11 plots. AF: allele frequency, CV: coefficient of variation, MAD: median absolute deviation, std: standard deviation. Statistical moments are calculated around mean.

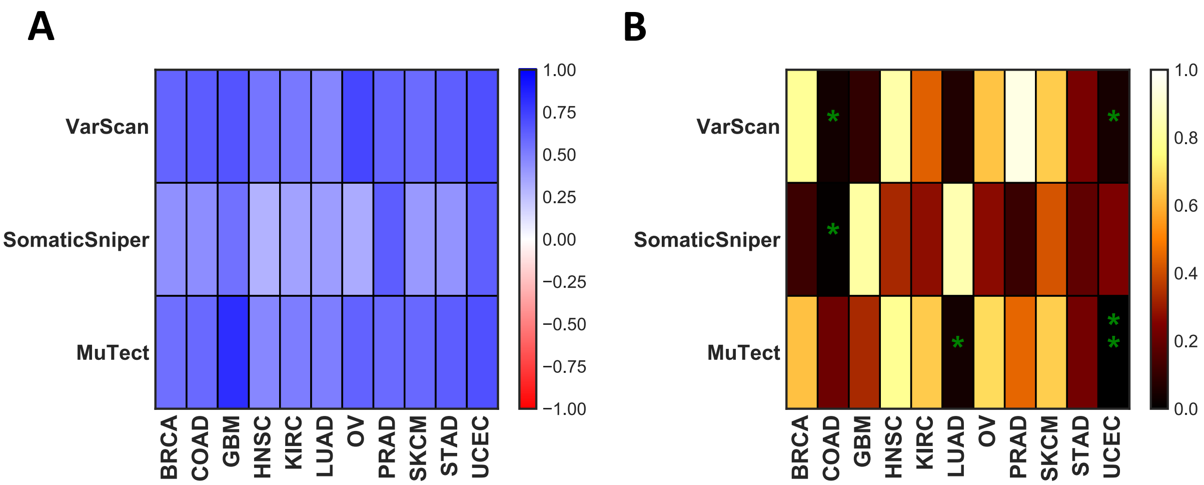

FIG. S18. **Supplementary Information.** Cancer cell fractions (CCF) an their prognostic evaluation A) Pearson correlation coefficient of CCF MATH score and allele frequency MATH score. B) Survival analysis log rank test p-values for high and low CCF MATH scores. Stars corresponds to values smaller than significance threshold 0.05. Double stars show significant results after Benjamini-Hochberg correction across all cancer types and mutation callers.

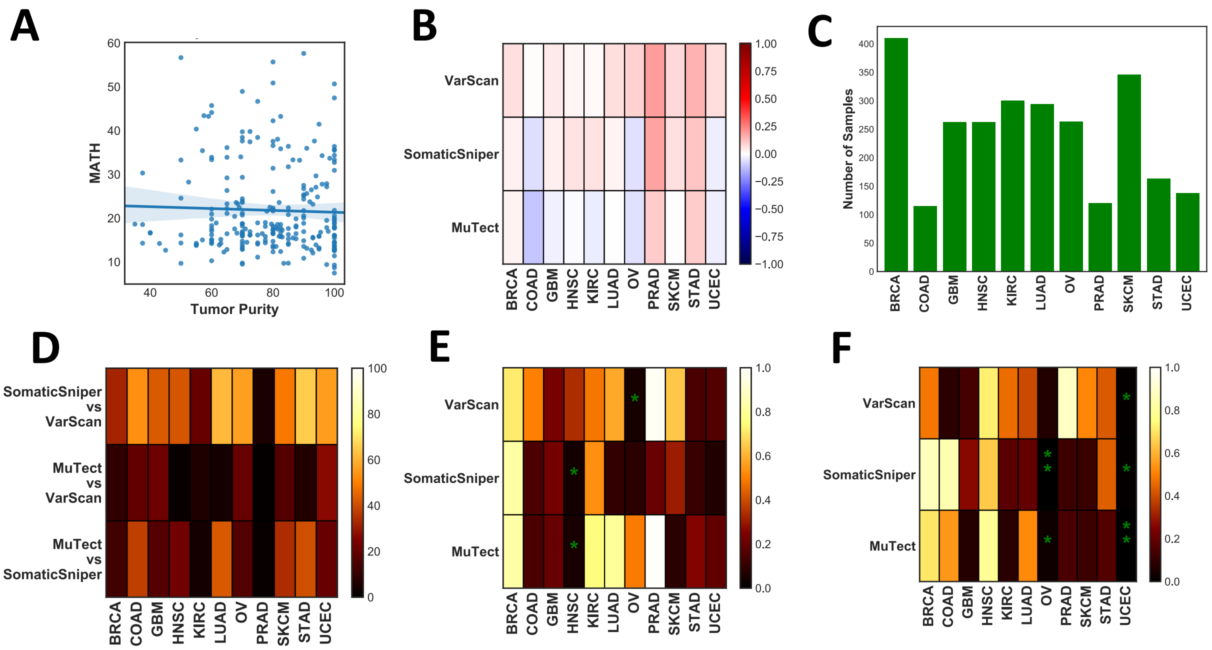

FIG. S19. **Supplementary Information.** Effect of tumor purity on allele frequency distributions. A) Plot of MATH against tumor purity for SNVs in UCEC called by MuTect, B) Pearson correlation coeficient between MATH and purity across cancers and mutation callers. If multiple samples were available for a patient, average tumor purity is used, C) Number of samples with purity greater than 0.8, D) Percentage of significantly different samples from subfigure C for pairs of mutation callers as determined by Kolmogorov-Smirnov test, E) Survival analysis log rank test p-values for high and low MATH score groups from samples in subfigure C, and F) similar analysis for CNV standard deviation. Stars corresponds to values smaller than significance threshold 0.05. Double stars show significant results after Benjamini-Hochberg correction across all cancer types and mutation callers.

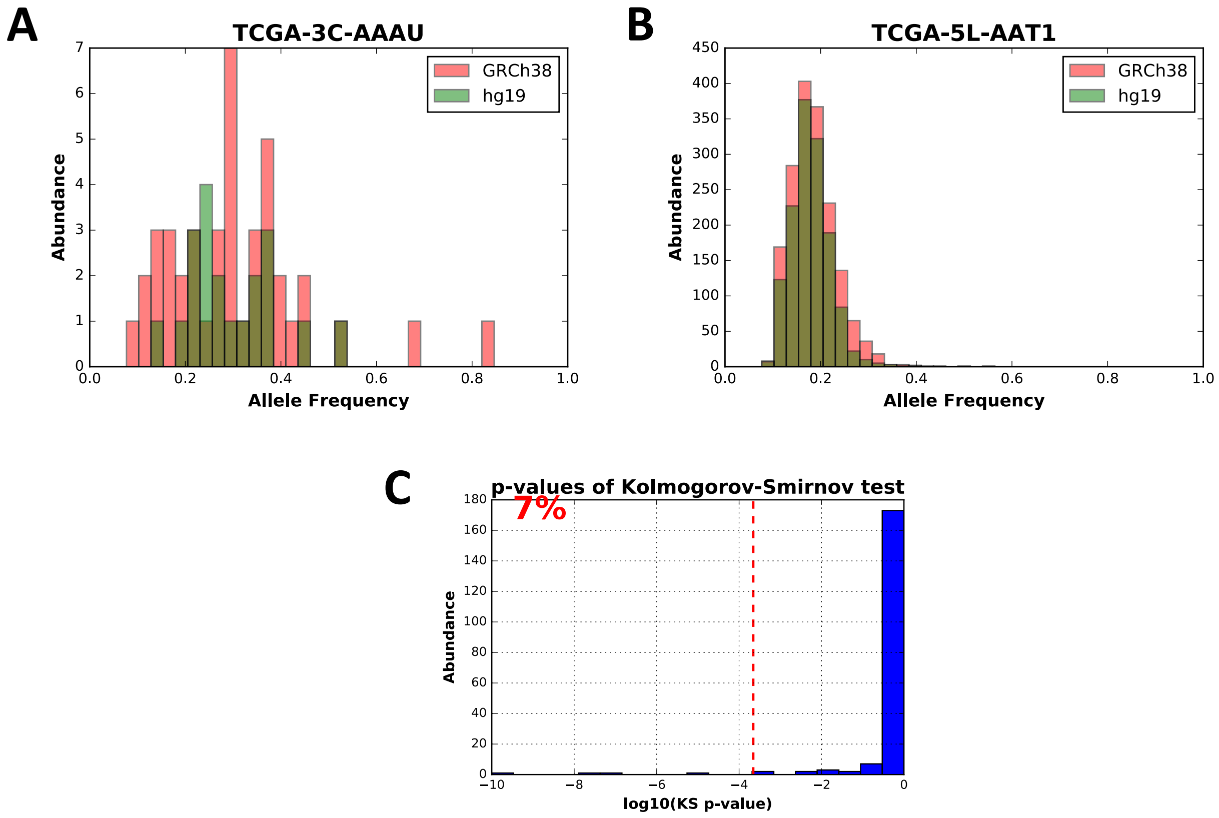

FIG. S20. **Supplementary Information.** A comparison of allele frequency distributions of some BRCA samples aligned to hg19 versus GRCh38. a,b) Two examples of allele frequency distributions. c) Distribution of p-values from the Kolmogorov-Smirnov test between the two alignments. 7% of the samples show significant differences according to Bonferroni corrected significance level 0.05

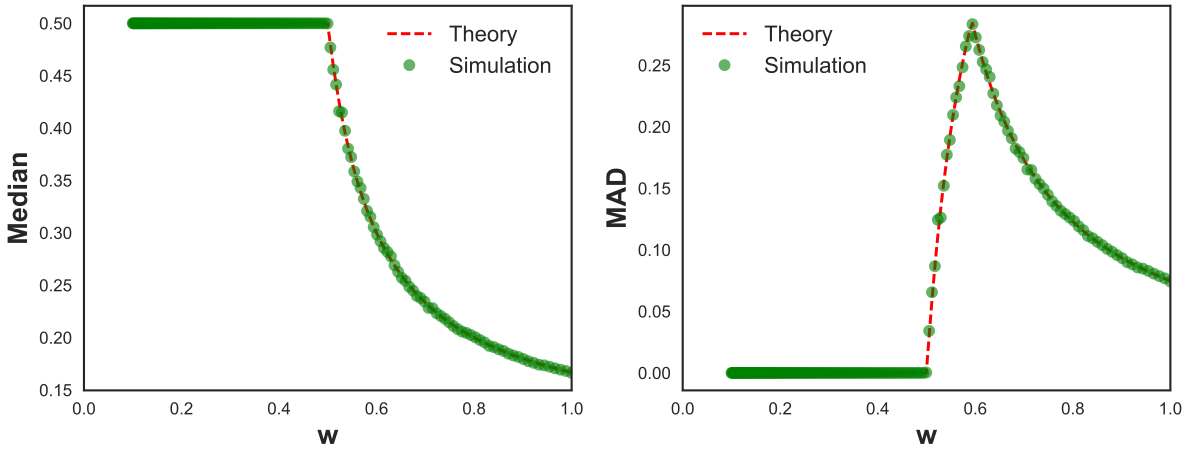

FIG. S21. **Supplementary Information.** Median and median absolute deviation (MAD) of the linear evolution model according to equations (6) and (15). Dashed red line is the theoretical result and green dots are calculated out of 100000 samples from the distribution in equation (3).

Mathematical Model

The mathematical model described in this paper is based on the linear evolution model of cancer [3]. Most mutations are neutral; however occasionally driver mutations occur which lead to fast selective sweeps. Allele frequencies are determined by the timing of the last selective sweep, and the history of tumor can be divided into two time periods in relation to this event. Consequently, we assume that there are two sets of somatic mutations in the tumor. The subclonal mutations which occurred after the last selective sweep, and the clonal mutations which occurred before the last selective sweep. The fraction of mutations in these two groups will be represented by w and $1-w$ respectively. The distribution of neutral subclonal mutations follows [4]:

$$P(F_N \le f) = \begin{cases} 0, \; f < f_{min}, \\ \beta\left(\alpha - \frac{1}{f}\right), \; f_{min} \le f \le 0.5. \end{cases} \tag{1}$$

where F_N is the random variable associated with allele frequencies of the neutral model. In order to have $P(F_N \le f_{min}) = 0$ and $P(F_N \le 0.5) = 1$, we set $\alpha = \frac{1}{f_{min}}, \beta = \frac{1}{\alpha - 2}$, where f_{min} is the minimum allele frequency measured. All the clonal mutations have allele frequency 0.5 and their distribution is:

$$P(F_C \le f) = \begin{cases} 0, & f_{min} \le f < 0.5, \\ 1, & f = 0.5. \end{cases} \tag{2}$$

where F_C is the random variable associated with clonal mutations. For allele frequency $F = F_N \cup F_C$, the overall probability distribution will be sum of probabilities (given that $F_N \cap F_C$=0) weighted by their rate of occurrence:

$$P(F \le f) = wP(F_N \le f) + (1-w)P(F_C \le f) \tag{3}$$

$$= \begin{cases} 0, & f < f_{min}, \\ w\beta\left(\alpha - \frac{1}{f}\right), & f_{min} \le f < 0.5, \\ 1, & f = 0.5. \end{cases}$$

To calculate MATH score for this distribution, we will calculate the median and median absolute deviation (MAD) separately.

Median

We will denote the median of allele frequencies by ϕ. In general ϕ has to satisfy the following inequalities:

$$\begin{cases} P(F \le \phi) & \ge 0.5, \\ P(F \ge \phi) & \ge 0.5 \end{cases} \implies \begin{cases} P(F \le \phi) & \ge 0.5, \\ P(F < \phi) & \le 0.5 \end{cases} \tag{4}$$

For $\phi = 0.5$ the first row of equation (4) is trivial ($1 \ge 0.5$), and the second row leads to $w \le 0.5$. On the other hand, for $f_{min} \le \phi < 0.5$, equation (4) reduces to:

$$P(F < \phi) = 0.5 \implies w\beta(\alpha - \tfrac{1}{\phi}) = 0.5 \tag{5}$$
$$\implies \phi = \tfrac{1}{\alpha - 1/\nu}$$

where $\nu = 2w\beta$, which leads to $\phi < 0.5$ only if $w > 0.5$. To summarize our results, the median of allele frequencies can be written as:

$$\phi = \begin{cases} 0.5, & w \le 0.5, \\ \frac{1}{\alpha - 1/\nu}, & w > 0.5. \end{cases} \tag{6}$$

Sampling from the distribution in equation (3) confirms this result (Figure S21).

Median Absolute Deviation (MAD)

We will denote MAD by m. It can be derived from the following relationships:

$$\begin{cases} P(|F-\phi| \le m) & \ge 0.5, \\ P(|F-\phi| \ge m) & \ge 0.5 \end{cases} \implies$$
$$\begin{cases} P(F \le \phi + m) - P(F < \phi - m) \ge 0.5, \\ P(F < \phi + m) - P(F \le \phi - m) \le 0.5 \end{cases} \tag{7}$$

If $w \le 0.5$ we have $\phi = 0.5$. We start by assuming that $m > 0$. In this case $\phi + m > 0.5$, leading to $P(F \le \phi + m) = 1$ and $P(F < \phi + m) = 1$. Hence equation (7) can be simplified to:

$$\begin{cases} P(F < \phi - m) \le 0.5, \\ P(F \le \phi - m) \ge 0.5 \end{cases} \tag{8}$$

Since $\phi - m < 0.5$ the functions are continuous and we can instead write $P(F \le \phi - m) = 0.5$. But $P(F \le \phi - m) \le P(F < 0.5) < w$ which cannot be true, given that $w \le 0.5$. As a result m cannot be positive. Since m is non-negative we conclude that $m = 0$.

On the other hand, for $w > 0.5$, we have $\phi = \frac{1}{\alpha - 1/\nu}$. In this case there are four possibilities for solving equation (7) that we will separately explore:

I. $\begin{cases} f_{min} \leq \phi + m < 0.5, \\ \phi - m < f_{min}. \end{cases}$

Functions are continuous in this region. So $P(F \leq \phi + m) = 0.5$, which can be solved similarly to equation (6) and gives $\phi + m = \phi \implies m = 0$. However this cannot be true, because the assumptions of this case can only be true for positive m.

II. $\begin{cases} f_{min} \leq \phi + m < 0.5, \\ f_{min} \leq \phi - m < 0.5. \end{cases}$

Functions are continuous in this region and we can write:

$$\begin{aligned} & P(F \leq \phi + m) - P(F < \phi - m) = 0.5 \qquad (9) \\ & \implies w\beta(\alpha - \frac{1}{\phi + m}) - w\beta(\alpha - \frac{1}{\phi - m}) = 0.5 \\ & \implies \frac{1}{\phi - m} - \frac{1}{\phi + m} = \frac{1}{\nu} \implies \frac{2m}{\phi^2 - m^2} = \frac{1}{\nu} \\ & \implies m^2 + 2\nu m - \phi^2 = 0 \\ & \implies m = -\nu + \sqrt{\nu^2 + \phi^2} \end{aligned}$$

This result is bounded by $m = 0.5 - \phi$.

III. $\begin{cases} 0.5 \leq \phi + m, \\ \phi - m < f_{min}. \end{cases}$

Functions are not continuous in this region. We can write the second row of equation (7) as

$P(F < \phi + m) \leq 0.5$. But $P(F < \phi + m) \geq P(F < 0.5) = w > 0.5$. Hence this cannot be true.

IV. $\begin{cases} 0.5 \leq \phi + m, \\ f_{min} \leq \phi - m < 0.5. \end{cases}$

Functions are not continuous in this region. We can write the first row of equation (7) as:

$$\begin{aligned} & P(F \leq \phi + m) - P(F < \phi - m) \geq 0.5 \qquad (10) \\ & \implies P(F < \phi - m) \leq 0.5 \\ & \implies 0.5\nu(\alpha - \frac{1}{\phi - m}) \leq 0.5 \\ & \implies \phi - m \leq \frac{1}{\alpha - \frac{1}{\nu}} \\ & \implies \phi - m \leq \phi \end{aligned}$$

which is a trivial result. For the second row of equation (7) we have:

$$P(F < \phi + m) - P(F \leq \phi - m) \leq 0.5 \qquad (11)$$

If $\phi + m > 0.5$ equation (11) leads to:

$$\begin{aligned} & P(F \leq \phi - m) \geq 0.5 \qquad (12) \\ & \implies 0.5\nu(\alpha - \frac{1}{\phi - m}) \geq 0.5 \\ & \implies \phi - m \geq \frac{1}{\alpha - \frac{1}{\nu}} \\ & \implies \phi - m \geq \phi \implies m = 0 \end{aligned}$$

which does not satisfy the assumptions of this case and cannot be true. On the other hand, if $\phi + m = 0.5$ equation (11) is equal to:

$$\begin{aligned} & P(F \leq \phi - m) \geq w - 0.5 \qquad (13) \\ & \implies 0.5\nu(\alpha - \frac{1}{\phi - m}) \geq w - 0.5 \end{aligned}$$

After some calculation we find:

$$w \leq 0.5 + \frac{\sqrt{\alpha^2 + 32} - \alpha}{16} \qquad (14)$$

In conclusion, only cases II and IV lead to acceptable solutions. To summarize these results, for MAD we have:

$$m = \begin{cases} 0, & w \leq 0.5, \\ 0.5 - \phi, & 0.5 < w \leq 0.5 + \Delta w \\ -\nu + \sqrt{\nu^2 + \phi^2}, & w > 0.5 + \Delta w. \end{cases} \qquad (15)$$

where $\Delta w = \frac{\sqrt{\alpha^2 + 32} - \alpha}{16}$. Sampling from the distribution in equation (3) confirms this result (Figure S21).

MATH Score

MATH can be derived by the following formula:

$$\text{MATH} = \frac{1.4826 \times m}{\phi} \times 100 \qquad (16)$$

where the constant is the scale factor for median absolute deviation. Using equations (6) and (15) we can write this as:

$$\text{MATH} = 148.26 \times \begin{cases} 0, & w \leq 0.5, \\ \frac{0.5}{\phi} - 1, & 0.5 < w \leq 0.5 + \Delta w \\ \frac{-\nu + \sqrt{\nu^2 + \phi^2}}{\phi}, & w > 0.5 + \Delta w. \end{cases} \tag{17}$$

[1] Li, Q.: Nonparametric testing of closeness between two unknown distribution functions. Econometric Reviews **15**(3), 261–274 (1996)
[2] Morris, L., Riaz, N., Desrichard, A., Şenbabaoğlu, Y., Hakimi, A.A., Makarov, V., Reis-Filho, J.S., Chan, T.A.: Pan-cancer analysis of intratumor heterogeneity as a prognostic determinant of survival. Oncotarget **7**(9), 10051–10063 (2016)
[3] Davis, A., Gao, R., Navin, N.: Tumor evolution: Linear, branching, neutral or punctuated? Biochimica et Biophysica Acta (BBA)-Reviews on Cancer **1867**(2), 151–161 (2017)
[4] Williams, M.J., Werner, B., Barnes, C.P., Graham, T.A., Sottoriva, A.: Identification of neutral tumor evolution across cancer types. Nature genetics **48**(3), 238–244 (2016)
